# No saturation in the accumulation of alien species worldwide

**DOI:** 10.1038/ncomms14435

**Published:** 2017-02-15

**Authors:** Hanno Seebens, Tim M. Blackburn, Ellie E. Dyer, Piero Genovesi, Philip E. Hulme, Jonathan M. Jeschke, Shyama Pagad, Petr Pyšek, Marten Winter, Margarita Arianoutsou, Sven Bacher, Bernd Blasius, Giuseppe Brundu, César Capinha, Laura Celesti-Grapow, Wayne Dawson, Stefan Dullinger, Nicol Fuentes, Heinke Jäger, John Kartesz, Marc Kenis, Holger Kreft, Ingolf Kühn, Bernd Lenzner, Andrew Liebhold, Alexander Mosena, Dietmar Moser, Misako Nishino, David Pearman, Jan Pergl, Wolfgang Rabitsch, Julissa Rojas-Sandoval, Alain Roques, Stephanie Rorke, Silvia Rossinelli, Helen E. Roy, Riccardo Scalera, Stefan Schindler, Kateřina Štajerová, Barbara Tokarska-Guzik, Mark van Kleunen, Kevin Walker, Patrick Weigelt, Takehiko Yamanaka, Franz Essl

**Affiliations:** 1 Senckenberg Biodiversity and Climate Research Centre (BiK-F), Senckenberganlage 25, 60325 Frankfurt am Main, Germany; 2 Department of Botany and Biodiversity Research, University of Vienna, Rennweg 14, 1030 Vienna, Austria; 3 Institute for Chemistry and Biology of the Marine Environment, University of Oldenburg, Carl-von-Ossietzky Strasse 9-11, 26111 Oldenburg, Germany; 4 Centre for Biodiversity and Environment Research, Department of Genetics, Evolution and Environment, University College London, Gower Street, London WC1E 6BT, UK; 5 Institute of Zoology, Zoological Society of London, Regent's Park, London NW1 4RY, UK; 6 School of Biological Sciences, University of Adelaide, Adelaide, South Australia 5005, Australia; 7 Centre for Invasion Biology, Department of Botany and Zoology, Stellenbosch University, Private Bag X1, Matieland 7602, South Africa; 8 Distinguished Scientist Fellowship Program, King Saud University, Riyadh 1145, Saudi Arabia; 9 Institute for Environmental Protection and Research (ISPRA), Via Vitaliano Brancati 48, 00144 Rome, Italy; 10 IUCN Species Survival Commission Invasive Species Specialist Group (ISSG), 00144 Rome, Italy; 11 Bio-Protection Research Centre, Lincoln University, PO Box 85084, Lincoln, Christchurch 7648, New Zealand; 12 Leibniz-Institute of Freshwater Ecology and Inland Fisheries (IGB), Müggelseedamm 310, 12587 Berlin, Germany; 13 Institute of Biology at the Department of Biology, Chemistry and Pharmacy, Freie Universität Berlin, Königin-Luise-Strasse 1-3, 14195 Berlin, Germany; 14 Berlin-Brandenburg Institute of Advanced Biodiversity Research (BBIB), Altensteinstrasse 34, 14195 Berlin, Germany; 15 IUCN Species Survival Commission Invasive Species Specialist Group (ISSG), University of Auckland, Auckland 1072, New Zealand; 16 Department of Invasion Ecology, Institute of Botany, The Czech Academy of Sciences, Zámek 1, CZ-252 43 Průhonice, Czech Republic; 17 Faculty of Science, Department of Ecology, Charles University in Prague, Viničná 7, CZ-128 44 Prague, Czech Republic; 18 German Centre for Integrative Biodiversity Research (iDiv), Halle-Jena-Leipzig, Deutscher Platz 5e, 04103 Leipzig, Germany; 19 Faculty of Biology, Department of Ecology and Systematics, School of Sciences, National and Kapodistrian University of Athens, Athens 15784 Greece; 20 Department of Biology, University of Fribourg, Chemin du Musée 10, 1700 Fribourg, Switzerland; 21 Department of Agriculture, University of Sassari, Viale Italia 39, 07100 Sassari, Italy; 22 CIBIO/InBIO, Centro de Investigação em Biodiversidade e Recursos Genéticos, Cátedra Infraestruturas de Portugal-Biodiversidade, Universidade do Porto, Campus Agrário de Vairão, 4485-661 Vairão, Portugal; 23 Zoologisches Forschungsmuseum Alexander Koenig, Museumsmeile Bonn, 53113 Bonn, Germany; 24 Department of Environmental Biology, Sapienza University, p. Moro, 5, 00185 Rome, Italy; 25 Department of Ecology, University of Konstanz, Universitätsstrasse 10, 78457 Konstanz, Germany; 26 Department of Biosciences, Durham University, South Road, Durham DH1 3LE, UK; 27 Departamento de Botánica, Facultad de Ciencias Naturales y Oceanográficas, Universidad de Concepción, Casilla 160-C, Concepción, Chile; 28 Charles Darwin Foundation, Puerto Ayora, Santa Cruz, Galápagos, Ecuador; 29 Biota of North America Program (BONAP), 9319 Bracken Lane, Chapel Hill, North Carolina 27516, USA; 30 CABI, Rue des Grillons 1, 2800 Delémont, Switzerland; 31 Department of Biodiversity, Macroecology and Biogeography, Georg-August-University Göttingen, Büsgenweg 1, 37077 Göttingen, Germany; 32 Helmholtz Centre for Environmental Research (UFZ), Department of Community Ecology, Theodor-Lieser-Strasse 4, 06120 Halle, Germany; 33 Department of Geobotany and Botanical Garden, Martin Luther University of Halle-Wittenberg, Am Kirchweg 2, 06108 Halle, Germany; 34 US Forest Service Northern Research Station, Morgantown, West Virginia 26505, USA; 35 Center for Interamerican Studies (CIAS), Department of Experimental and Systems Ecology, Bielefeld University, Universitätsstrasse 25, 33615 Bielefeld, Germany; 36 Botanical Society of Britain and Ireland (BSBI), Suite 14, Bridge House, 1-2 Station Bridge, Harrogate HG1 1SS, UK; 37 Department of Biodiversity and Nature Conservation, Environment Agency Austria, Spittelauer Laende 5, 1090 Vienna, Austria; 38 Department of Botany, National Museum of Natural History, MRC-166 Smithsonian Institution, P.O. Box 37012, Washington, DC 20013, USA; 39 Institut National de la Recherche Agronomique (INRA), UR 0633, Zoologie Forestière, 45075 Orléans, France; 40 Centre for Ecology and Hydrology, Maclean Building, Benson Lane, Crowmarsh Gifford, Wallingford OX10 8BB, UK; 41 IUCN Species Survival Commission Invasive Species Specialist Group (ISSG), Via Valentino Mazzola 38 T2 B 10, I-00142 Roma, Italy; 42 Faculty of Biology and Environmental Protection, Department of Botany and Nature Protection, University of Silesia, Jagiellonska Strasse 28, 40-032 Katowice, Poland; 43 Institute for Agro-Environmental Sciences, NARO (NIAES), 3-1-3 Kannondai, Tsukuba 305-8604, Japan

## Abstract

Although research on human-mediated exchanges of species has substantially intensified during the last centuries, we know surprisingly little about temporal dynamics of alien species accumulations across regions and taxa. Using a novel database of 45,813 first records of 16,926 established alien species, we show that the annual rate of first records worldwide has increased during the last 200 years, with 37% of all first records reported most recently (1970–2014). Inter-continental and inter-taxonomic variation can be largely attributed to the diaspora of European settlers in the nineteenth century and to the acceleration in trade in the twentieth century. For all taxonomic groups, the increase in numbers of alien species does not show any sign of saturation and most taxa even show increases in the rate of first records over time. This highlights that past efforts to mitigate invasions have not been effective enough to keep up with increasing globalization.

## Abstract (web summary)

Alien species of animals and plants can invade new regions of the earth. This study performs a global analysis of temporal dynamics and spatial patterns of alien species introductions over the past 200 years, and reports no saturation in the rate at which these invasion are increasing.

The rate at which humans translocate species beyond their native ranges has substantially increased during the last centuries^1^,^2^,^3^. The unprecedented intensity of human-mediated species exchange leads to the homogenization of floras and faunas^4^, re-defines the classical boundaries of biogeography^5^ and has far-reaching implications for native biota, ecosystem functioning, human health and economy^6^,^7^,^8^. However, although the general rise in the number of alien species is undisputed, we know little about the temporal dynamics of alien species accumulation and how this might vary among taxonomic groups and geographic regions.

A wide range of motivations underlie the introduction of alien species and the relative importance of these vary considerably in space and time, and among taxonomic groups^9^. For example, many alien species of taxa such as mammals, plants or birds were released by European explorers and settlers all over the world during 1500–1800 (ref. 9). In the nineteenth century, numerous plants have been brought to Europe for ornamental purposes^10^, whereas currently bird species are intensively traded in South East Asia, where the accidental or deliberate release of individuals supports the establishment of alien populations^3^. In addition, most alien species of taxa, such as insects, algae and crustaceans, have been introduced recently through trade and the transport of goods and people^11^. Differences in the pathways and distribution of alien species introductions suggest that the chronology of invasion probably varies among taxonomic groups and regions, yet a comprehensive analysis of global invasion dynamics of the last centuries is still lacking. Furthermore, we have as yet only limited understanding of whether current rates of alien species accumulation may show signs of saturation or whether we can expect biological invasions to continue at the same rate seen in the past.

To address these gaps in our knowledge, we compiled a global data set of regional first records of alien species that are now established (following the criteria in ref. 12) in multiple geographic regions worldwide (countries and sub-national regions such as islands). This data set of 45,813 first records of 16,926 established alien species from a wide range of taxonomic groups is invaluable for assessing taxonomic and geographic variation in alien species accumulations, and for testing for evidence of slowdown in the accumulation rates. It covers 282 non-overlapping regions from all continents, with particularly intense sampling in Europe, North America and Oceania, and from well-studied taxa such as vascular plants, mammals, insects, birds and fishes (Fig. 1 and Supplementary Fig. 1). This data set allows us to analyse variations in the rate of alien species introductions across space and time in a consistent way over large geographic scales. In particular, we test the following predictions: (1) rates of introductions for species often intentionally introduced such as mammals, birds and vascular plants should decline in recent years as a result of increased awareness of their impacts and tighter biosecurity regulations; (2) rates of introductions for taxonomic groups primarily introduced accidentally such as invertebrates or algae should show steep increases in recent times, as these species are more difficult to regulate and are closely associated with increasing trade; and (3) significant geographic differences in the rates of alien species introductions should be apparent, reflecting variations in socio-economic histories and the strength of biosecurity regulations. We find that the number of established alien species and for most taxonomic groups even the rate of introduction increased until recently with no sign of saturation. We can therefore expect many more invasions to happen in the near future.

## Results

### Global patterns of alien species introductions

**Figure 2:**
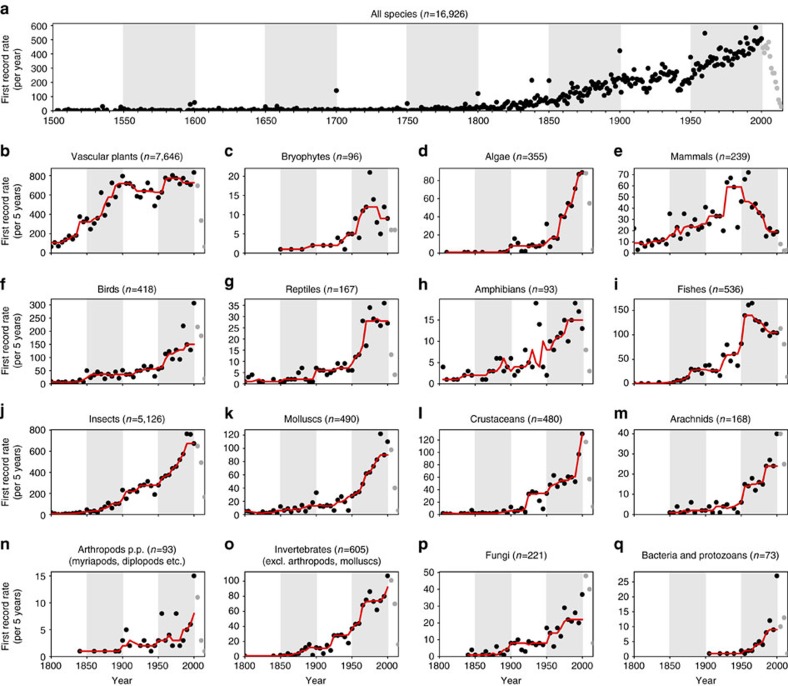
Global temporal trends in first record rates. Global temporal trends in first record rates (dots) for all species (**a**) and taxonomic groups (**b**–**q**) with the total number of established alien species during the respective time periods given in parentheses. Data after 2000 (grey dots) are incomplete because of the delay between sampling and publication, and therefore not included in the analysis. As first record rates were recorded on a regional scale, species may be included multiple times in one plot. (**a**) First record rates are the number of first records per year during 1500–2014. (**b**–**q**) First record rates constitute the number of first records per 5 years during 1800–2014 for various taxonomic groups. The trend is indicated by a running median with 25-year moving window (red line). For visualization, 50-year periods are distinguished by white/grey shading.

The global rate of first records (measured as the number of first records of established alien species per time unit) remained low between 1500 and 1800 (on average 7.7 first records annually, Fig. 2a). Since 1800, first records have increased constantly, only slowing during the two World Wars, to a maximum of 585 in 1996 (reflecting on average more than 1.5 new records per day). Our data set does not cover all alien species recorded in every region of the world and thus inevitably underestimates first record rates. The continuous rise in first record rates during the last 200 years is consistent across taxa (Fig. 2), except for mammals and fishes, whose rates have declined in recent decades (Fig. 2e,i). Remarkably, barring mammals and fish, there is no clear indication of a slowdown in the first records rates of alien species: rather, they are still increasing. This trend was consistent for both mainlands and islands (Supplementary Fig. 2).

**Figure 3:**
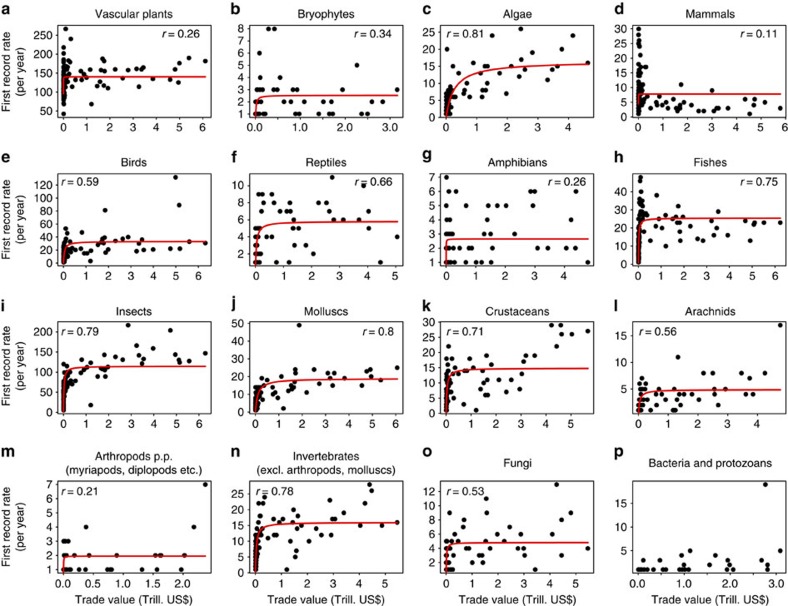
Relationships between the values of annually imported commodities and first record rates. Relationships between the values of annually imported commodities and first record rates of the same regions for all taxonomic groups separately (**a**–**p**). Each dot represents a single year during 1870–2000, depending on data availability. Following previous studies^20^,^21^, a Michaelis–Menten curve (lines) was fitted to test for an improved fit using a nonlinear relationship with an attenuation of first record rates at large import values. The goodness-of-fit between observed data and the fitted curve is indicated by the Pearson's correlation coefficient given in the upper left corner of sub-panels, except for bacteria and protozoans, where the fitting function did not converge.

Three general patterns emerge in the first record trajectories of alien species. The first pattern consists of weak increases until *ca*. 1950, followed by strong increases thereafter (Fig. 2) and is best described by an exponentially increasing function of time (Supplementary Fig. 3). This pattern is typical of species mainly introduced accidentally as stowaways on transport vectors or contaminants of commodities (for example, algae, insects, crustaceans, molluscs and other invertebrates; Fig. 2d,j–l,o). Indeed, first record rates of these taxa are highly correlated with the trade values of imported commodities of the respective countries (all correlation coefficients>0.71; Fig. 3c,i–k,n and Supplementary Fig. 4). Similar patterns, although with lower correlation coefficients, were observed for birds and reptiles (Figs 2f,g and 3e,f). Many of these taxa have been introduced intentionally as pets, particularly in recent times^13^, increasing the likelihood of the establishment of alien populations. First record rates produced by a simple neutral colonization model that assumes that the probability of introduction increased proportional to the value of imported commodities showed rates similar to those observed for most invertebrates and algae, with a steep increase after 1950 (Supplementary Fig. 5j–l). This finding supports the suggested importance of trade as a major driver of alien species introductions at least for invertebrates and algae.

**Figure 4:**
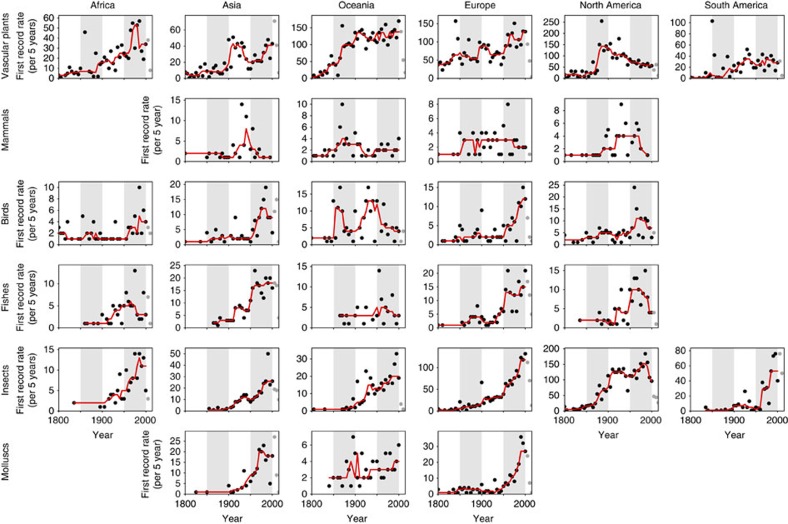
Temporal trends in continental first record rates. Temporal trends in continental first record rates (that is, first records of established alien species on a continent per 5 years, dots) for various taxonomic groups and continents (for delineation of continents see Supplementary Fig. 12). It is noteworthy that for the inter-continental comparison, we only considered first records of established alien species on a continent, to avoid a bias due to varying numbers of countries in a continent, which resulted in a reduced number of first records (56% of the full data set). The trends are indicated by a running median with 25-year moving window (red line). Data after 2000 (grey dots) are incomplete and were removed from analysis. Time series with <70 first records are not shown. For visualization, 50-year periods are distinguished by white/grey shading. Time series for taxonomic groups with low numbers of first records are shown in Supplementary Fig. 6.

Second, first record rates of mammals and fishes increased until around 1950, then declined subsequently (Fig. 2e,i), and were best fitted by a hump-shaped function of time (Supplementary Fig. 3d,h). A similar pattern can also be found for the time series of other taxonomic groups after taking into account most recent first record rates (>2000, that is, including grey dots in Fig. 2). However, these recent data are likely to be influenced by a reduced sampling intensity due to delays in detection and reporting of new alien species and thus should be interpreted very carefully. A longer sampling time and more recent data are needed to reliably assess these trends. For mammals, the findings are in line with our expectations that the rates of first records of species mostly introduced intentionally should decline in recent years. Despite the observed declines, first record rates are still high with 19 first records for mammals and 104 for fishes during 1996–2000 (Fig. 2). The decline seemed to result from a reduction in the deliberate introduction of mammals as, for example, game animals or for the fur industry, and stricter regulations for animal farming, which resulted in fewer escapes than was historically the case. For instance, Acclimatization Societies founded after 1860 in the United States, Australia and New Zealand were responsible for introductions of numerous mammals, birds and plants^9^,^14^,^15^. In the twentieth century, their activity in these regions declined due to decreasing public and scientific support^15^. The decrease in first record rates may also be related to changes in manner and to stricter regulations of animal farming, which should result in fewer escapes. Some of these explanations may also apply to fishes; in Europe, however, first record rates of fishes have increased continuously (Fig. 4), which may be at least partly attributed to the ongoing immigration of new fish species through the Suez Canal^16^.

Third, first record rates of vascular plants increased steeply in the nineteenth century and remained at high levels until the present (Fig. 2b), which was best represented by a sigmoidal increase of first record rates with time (Supplementary Fig. 3a). This trend can again be at least partly attributed to the colonization of North America and Oceania by European settlers and corresponding activities of institutions such as Acclimatization Societies in the nineteenth century. In addition, the foundation of many botanic gardens worldwide, a major pathway for plant introductions^17^, together with the increased international transport of living plants and propagules, and inventions such as the Wardian Case in 1829 (a mobile greenhouse to transport live plants), have promoted the establishment of alien plant species. In contrast to mammals and fishes, and contrary to our expectations, the first record rates of vascular plants remained high in the twentieth century, which is likely to be a consequence of the intensification of global trade^18^ and increasingly widespread cultivation of plants in agriculture, botanic and private gardens^17^.

Temporal patterns in the rates of first records on a continent (thereby excluding subsequent within-continental spread) vary distinctly across continents and taxonomic groups (Fig. 4 and Supplementary Fig. 6). Within taxonomic groups, there may be different pathways driving invasions of species that differ in their ecology and temporal patterns in these pathways are likely to vary among world regions. Indeed, we did not find a consistent pattern in the temporal trends within a taxonomic group across continents (Supplementary Fig. 7). Some of the observed inter-continental variation seems to be a consequence of European colonization, such as the steep increase of first record rates of alien vascular plants in the nineteenth century in North America and Oceania (Fig. 4). However, most of the inter-continental variation is difficult to explain, due to the lack of knowledge and data of the underlying processes, and the high inter-annual variation of the first record rates. Remarkably, most of these temporal variations in first record rates are nearly impossible to detect using cumulative numbers of alien species (Supplementary Fig. 8), a common way of presenting alien species accumulations.

The first record of an alien species may be the result of a human-mediated introduction of that species into a region, or a consequence of previous introductions into neighbouring areas and subsequent natural spread into adjacent regions. To remove the influence of introductions due to the species natural range expansion in the alien range, we considered only the first records of alien species on a continent, which revealed qualitatively similar, although less clear, trends compared with the full data set (Supplementary Fig. 9). First records are often influenced by a time lag between the actual introduction of a species and its detection^19^. The delay is likely to have decreased with time due to more intense sampling (for example, national species inventories) in recent decades, resulting in earlier detections of alien species after their introduction into new regions. However, the chronology of first record rates within the same taxonomic group should not change qualitatively (see Supplementary Notes for a detailed assessment of data quality and a discussion of the potential influence of varying sampling intensity on study results).

### Predicting alien species accumulations

Trade is consistently reported as a crucial predictor for the number of alien species in a country^6^,^18^,^20^. Although these studies used total alien species numbers, we here test the congruence of the temporal development of import values and alien species accumulations. The relationship between alien species numbers and the values of imported commodities was found to be nonlinear, best described by a function saturating at large import values^20^,^21^. This was confirmed for some taxonomic groups (for example, algae, insects and molluscs), but not for others (vascular plants, mammals, fishes and crustaceans; Fig. 3).

**Figure 5:**
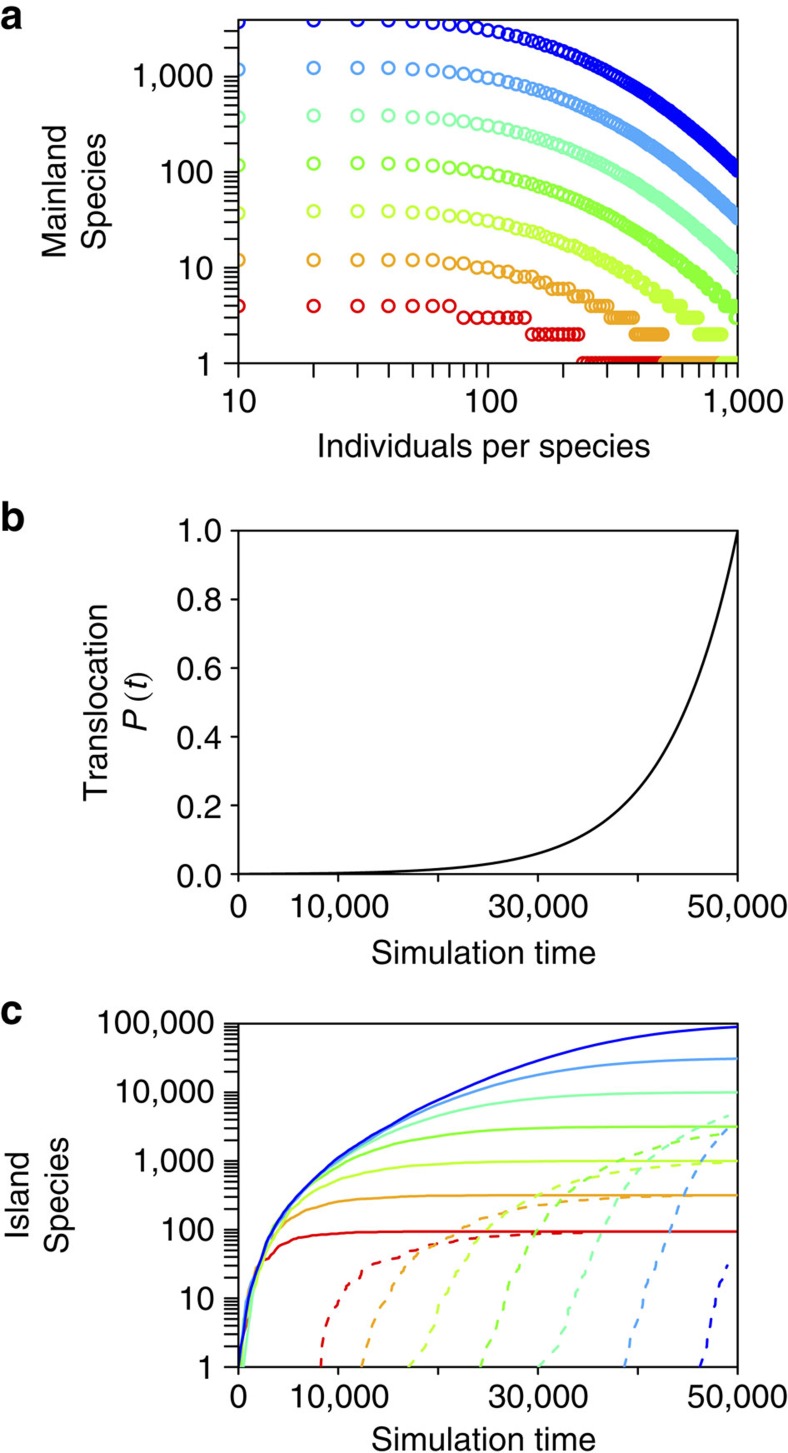
Simulation results for the accumulation of alien species on an island. (**a**) Nine arbitrary mainland communities (colours) with log-normally distributed species numbers ranging from *n*=100 (red) to *n*=100,000 (blue) species were considered. (**b**) At each simulation time step, a propagule from a mainland community was selected with time-dependent probability *P*(*t*), which exponentially increased with simulation time *t*, shown in **b** and translocated to the island (see Supplementary Fig. 5 for the results using different shapes of *P*(*t*)). (**c**) The resulting accumulation of species numbers on the island (solid lines) shows that the timing of saturation highly depends on the size of the mainland community. Considering an Allee effect in the model, expressed as a certain number of propagules (here >10 propagules) necessary to establish an alien population during a given time period, delayed the accumulation of species (dashed lines), but did not change the results qualitatively. The lowest probability for establishment on the island is given by a low probability of translocation of an individual, which is randomly depicted from the mainland community, and a high species richness of the mainland community (blue lines): in the rare case of translocation, chances are high that an individual of a new species will be selected, which resulted in low population sizes of the same species on the island and a high chance of going extinct due to the Allee effect. This resulted in a distinct delay of the accumulation of established alien species on the island when the mainland community consists of many species with high abundances (blue dashed line).

In theory, the accumulation of species should slow down at some time, for example, due to the depletion of incoming species pools or regional saturation. Knowledge about the future development of alien species accumulation would be important for management strategies to counteract new alien species introductions and impacts. The results of our model indicate that the prediction of future trajectories of alien species accumulation highly depends on the size of the pool of potential invaders (Fig. 5) and how the probability of introduction changes with time (Supplementary Fig. 5). Introducing other mechanisms relevant for the establishment of alien populations, such as an Allee effect, delayed the process of invasion, but revealed qualitatively similar results (dashed lines in Fig. 5c). The pool of potential invaders consists of those species, which are capable of being introduced into the focal region and establish an alien population. This species pool results from the complex interplay of species' native distributions, their abundances in the native range, environmental matches and colonization and propagule pressure, which further depends on the pathways connecting native and focal region and their rates of transport (for example, import volumes)^5^,^22^,^23^,^24^. All of these factors are likely to have changed during the last centuries. Although it may be possible to dissect the interactions of these factors in case studies of individual guilds of species in specific regions, such an analysis is nearly impossible to perform on a global scale. Yet, without a thorough knowledge about the underlying mechanisms driving past invasion dynamics, it will be very difficult to determine trajectories of future alien species accumulations other than extrapolating fitted trends, which are, however, associated with high uncertainty (but see ref. 18 when time lags are involved).

## Discussion

We show for the first time at a global scale that the increase in numbers of alien species does not show any sign of saturation. For most taxa, even the rate of first records increased over time with highest rates of first records being observed in recent times. Likewise, the implementation of national legislation and international agreements aiming to reduce alien species threats to biodiversity, economy and human well-being has also distinctly increased during the last 100 years^25^ and, without these, the number and impact of alien species would have probably been much worse. For example, the rates of alien insects decreased for certain feeding guilds in the United States likely to be as a consequence of the implementation of the Plant Quarantine Act in 1912 (ref. 26). However, the continuous increases in the rates of alien species first records show that these regulations have not been effective enough to keep up with increasing rates of global trade and slow down alien species accumulation, especially those arriving mainly accidentally, such as invertebrates and pathogens. An exception is the Biosecurity Act in New Zealand adopted in 1993, which represents the most comprehensive and stringent national law for the prevention of alien species introductions currently in force. Consequently, first record rates of vascular plant species in New Zealand clearly dropped in the 1990s (Supplementary Fig. 10). This probably reflects the strength of using a white-list of permitted species, which requires any unlisted species to be fully risk assessed before entry into the country is allowed. Most other comprehensive national regulations now in force, and constantly evolving (for example, in Japan, Australia, South Africa, the United States and the European Union), are based on blacklists of unwanted species. The effectiveness of these procedures needs further research and improvements are desirable^22^,^27^, the more as many recently introduced alien species were not known as problematic in their country of origin, or even unknown to Science. Assessments of specific biosecurity regulations require more detailed analyses than are possible in this study, due to its broad geographic and taxonomic scope.

The lack of saturation in the global accumulation of alien species has important implications for understanding biodiversity patterns. Certainly, extinctions (irrespective of the influence of alien species) have increased in recent times, but at lower rates than first records of alien species. Consequently, the net numbers of species in most regions have increased over time^4^,^28^,^29^. This may indicate that regional species pools are unsaturated^30^,^31^. However, such an increase in species numbers may be a transient phenomenon (for example, due to extinction debt^32^) and thus new levels of species richness—which will then consist of fewer native and more established alien species—may only be reached in the long-term. New levels of regional species richness are expected to be higher than those witnessed in the past, but these increases would come at the cost of a variety of impacts on native ecosystems^7^, the global homogenization of floras and faunas^4^,^5^, and the global extinction of native biota. This is of particular concern on islands, where impacts of established alien species are strongest and many native species are endemic^33^. Thus, although introductions of alien species may increase regional species richness, they will continue to decrease global species richness^28^ and β-diversity^4^,^5^.

The continuous increase in first record rates suggests that the numbers of new alien species will most probably further increase, as current tools to prevent biological invasions are not effective enough to slow down the ever-increasing alien species numbers. The pathways by which alien species are introduced into new areas are also changing rapidly, in particular through increased global trade, tourism, agriculture, horticulture, and the construction and formation (for example, through climate change) of new transportation corridors, such as the opening of the Arctic Ocean shipping routes^22^,^34^. Future threats due to alien species may be greatest in emerging economies due to these factors^18^,^22^. Although deleterious impacts caused by alien species have been recognized widely in legislation^25^, there is an urgent need to implement more effective prevention policies at all scales, enforcing more stringent national and regional legislations, and developing more powerful international agreements. As highlighted by the mid-term analysis of the Strategic Plan for Biodiversity 2020 (refs 2, 35), current efforts are still largely inadequate to reduce the accumulation of alien species.

## Methods

### Data compilation

Data on first year of record of established alien species were gathered from various sources including online databases, scientific peer-reviewed publications, reports, books and personal collections (Supplementary Table 1). We standardized scientific names of species with global checklists or adopted taxonomic views provided by global databases. For example, vascular plant species names were standardized in accordance with ‘The Plant List' (www.theplantlist.org), which offers the most comprehensive global taxonomic reference for plants, using functions provided in the R^36^ package ‘Taxonstand'^37^ and for avifauna we adopted the taxonomy of the Global Avian Invasions Atlas^38^, which provides most first records of birds in the database (>90%). In case of multiple entries of the same species in the same region, only the first record was kept. Although removing these duplicated entries, preferences were given to high-quality sources and large databases with a high degree of standardization, such as DAISIE (www.europe-aliens.org/), CABI Invasive Species Compendium (www.cabi.org/isc/) or GAVIA^38^ or peer-reviewed scientific publications.

The year of first record was provided in the vast majority of cases as a single year (87% of all records). If time periods were provided such as 1940–1950 or ‘1940s', a year within the respective range was randomly selected to avoid arbitrary peaks at, for example, the mean value of the ranges. Data with time periods longer than 20 years such as ‘first half of nineteenth century' were not considered for the analysis. If only the last year of a range was given such as ‘<1980', the respective year was taken as the first record. Finally, the database contained 55,099 first records of 19,031 introduced alien species. The status of invasion (casual or established) was assessed based on information provided in the original source. To exclude ephemeral alien species, casuals were removed from the analysis. We restricted the analysis to first records after 1500 due to data availability, which resulted in a total of 45,813 first records for 16,926 established species.

Region names were standardized to obtain a unique set of 282 non-overlapping regions (countries and sub-national regions such as islands). In addition, we included a data set of alien insect first records only available for the United States and Canada combined. In case of duplicated entries from different sources, we preferred the information provided for the individual country (United States or Canada) and removed the entry from the combined data set. In most cases, the names of the countries were adopted. Only islands politically belonging to a mainland country within another climatic or biogeographic region or with high quality data of first records were considered independently. For example, the Hawaiian Islands and Puerto Rico were distinguished from the United States, Galápagos from Ecuador, Tasmania from Australia, La Réunion from France, Azores from Portugal and Corsica from France. Small islands close to the mainland country were not considered separately.

Data of bilateral trade between countries were taken from the ‘Correlates of War' project^39^. This data set provides bilateral trade data in current US$ between 1870 and 2009. The number of countries for which trade data were available increased with time from 27 countries in 1870 to 185 countries in 2000. To test for potential influences of the lack of deflation of trade data, we corrected the value of imported commodities by dividing by the Consumer Price Index (CPI) of the same year. As the CPI is only available during a limited time period, we tested two CPI data sets of varying resolution: (1) CPI of 183 countries during at longest 1969–2000 obtained from the United States Department of Agriculture Economic Research Service (www.ers.usda.gov) and (2) CPI of the USA spanning a longer period (1913–2000) provided by the US Bureau of Labor Statistics (available at www.inflationdata.com). The latter was taken for all countries. The year of foundation of botanic gardens were obtained from the online database of the Botanic Gardens Conservation International (www.bgci.org). This database contains 1,633 entries of the year of foundation of botanic gardens in 150 countries worldwide. All maps were created using the delineation of countries provided in the freely available ‘maptools' package^40^ of the open source software R^36^. Coordinates of islands were extracted from the same package and supplemented with data from Weigelt *et al*.^41^.

### Statistical analysis

The statistical analysis was restricted to first records until the year 2000, to avoid a potential bias due to lags in the recording of new alien species. To test for different shapes of the temporal trends of first record rates, we fitted five functions to the time series' of first record rates: linear [*y*=*a*+*bx*], exponential [*y*=*a*e^*bx*^], saturating [*y*=*a*(1-e^−*bx*^)] and sigmoidal [*y*=*a*(*x*^*b*^/(*x*^*b*^+*c*^*b*^))] increase of first record rates *y* with time *x*, with *a*, *b* and *c* denoting constants. In addition, a Weibull distribution was fitted to test for a potential decline in recent years. The functions were fitted individually to the time series' of first record rates using the Nelder–Mead optimization algorithm implemented in the ‘optim' function in the statistical software environment R^36^. The algorithm tries to reduce the deviation between observed and predicted data by minimizing Akaike's Information Criterion. After fitting each function individually, the one which describes the time series' of first record rates best was selected by the smallest Akaike's Information Criterion.

To investigate the relationship between the values of imported commodities and first record rates, a correlation analysis between the temporal developments of the annual values of imported commodities in current US$ of a country and the temporal dynamics of first record rates was performed. The relationship between import values and alien species introductions is assumed to be nonlinear, saturating at large import values^20^,^21^. Thus, a Michaelis–Menten curve was fitted, which is described as *R*=*R*_max_*M*/(*K*+*M*), with *R* denoting the first record rate, *M* the annual value of imported commodities, and *R*_max_ and *K* being constants. This model produces a convex (saturating) curve; it was previously applied to model cumulative species numbers as a function of cumulative trade^20^,^21^, but here we apply the model to non-cumulative values. Trade data were available only during 1870–2000 and for countries; thus, correlation analysis was only performed for those times and countries with available data of import values and first record rates. The analysis was repeated using deflated and non-deflated import values for the respective time periods to test for the potential influence of using non-deflated import values. As the differences were marginal (Supplementary Table 2), we used the non-deflated import values because of the longer time period available.

### Model description

A simple colonization model was developed to investigate the influence of a temporally variable introduction rate on the accumulation of alien species. The model is based on ideas from Neutral Theory^42^, assuming that all propagules of the community have the same chance of being translocated and establish a new population.

Consider an arbitrary mainland community with *M* species and *I* propagules drawn from a log-normal distribution (for example, Supplementary Fig. 5a). At each simulation time step *t*, a propagule *i* of the mainland community was randomly selected with time-dependent probability *P*(*t*) and added to a new habitat, the island. With probability (1−*P*(*t*)) no propagules were introduced and the communities on the mainland and island remain unaffected. In the basic model, the introduction of a single propagule resulted in the establishment of that species on the island. This may be unrealistic for some taxonomic groups and species and thus we extended the model by incorporating an Allee effect: a population is considered to become established on the island only if the number of propagules of the same species found on the island during a time period *T* is above the Allee threshold *a*. As an increase in *a* is equivalent to a decrease in *T*, we set *T* arbitrarily to 1,000 simulation time steps and varied *a*. The consideration of an Allee effect delayed the accumulation of alien species, but did not change the model results qualitatively. Increasing *a* delayed the invasion process further without distinctly affecting the overall results (Supplementary Fig. 11).

The island was assumed to be large enough to avoid effects of saturation of species/propagules due to size limitation of the island. This may be regarded as a strong assumption, but it is supported by various studies indicating that none of the investigated regions around the world were saturated with species^30^,^43^,^44^. In different scenarios, the time dependence of *P*(*t*) was chosen to stay constant or to increase with simulation time with different shapes (linear or exponential, Supplementary Fig. 5). *P*(*t*) was also considered to increase proportionally to the temporal development of the global annual value of imported commodities or the cumulative number of newly founded botanic gardens. For the comparison of different temporal developments, *P*(*t*) was standardized that on average the same number of propagules arrived on the island during the full simulation time. The temporal developments of the species on the island were shown either as the number of new species recorded on the island during a defined time period (that is, as first record rate, Supplementary Fig. 5) or as cumulative species numbers (Fig. 5) (see Supplementary Notes for a detailed discussion of model results). All data analyses and modelling were performed using R^36^. The R code for the implementation of the model is freely available online (www.dx.doi.org/10.12761/SGN.2016.01.022).

### Data availability

Annual numbers of first records of taxonomic groups and continents and R code of the implementation of the invasion model are freely provided online (www.dx.doi.org/10.12761/SGN.2016.01.022).

## Additional information

**How to cite this article**: Seebens, H. *et al*. No saturation in the accumulation of alien species worldwide. *Nat. Commun.* **8**, 14435 doi: 10.1038/ncomms14435 (2017).

**Publisher's note**: Springer Nature remains neutral with regard to jurisdictional claims in published maps and institutional affiliations.

## Supplementary Material

Supplementary Information Supplementary Figures 1-12, Supplementary Tables 1-2, Supplementary Note 1 and Supplementary References

## Figures and Tables

**Figure 1:**
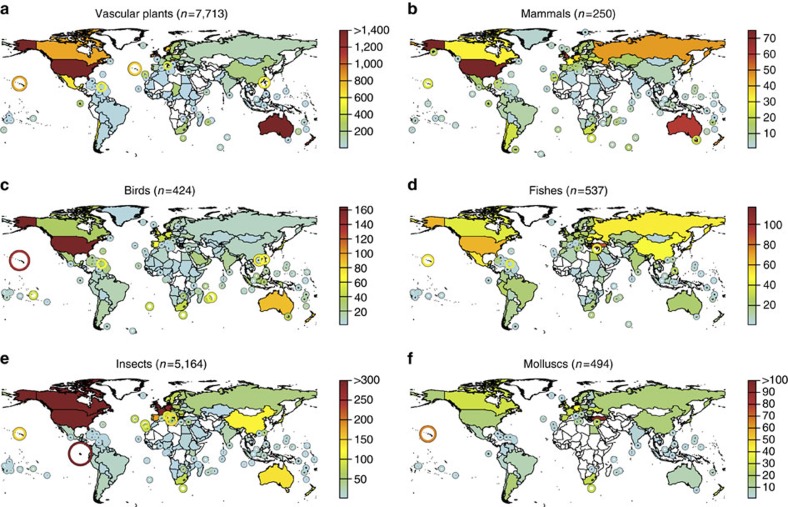
Number of first records of established alien species per region (mainlands and islands) for major taxonomic groups. (**a**–**f**) Colour and size of circles indicate the number of first records of established alien species. Circles denote first records on small islands and archipelagos otherwise not visible. The world maps were created using the ‘maptools' package^40^ of the open source software R^36^.
