# Supplementary material for: No saturation in the accumulation of alien species worldwide
Source: Nat Commun. 2017 Feb 15;8:14435. doi: 10.1038/ncomms14435 (PMC5316856; doi:10.1038/ncomms14435)
Supplement: Supplementary Information — Supplementary Figures 1-12, Supplementary Tables 1-2, Supplementary Note 1 and Supplementary References [file ncomms14435-s1.pdf]

Supplementary Figures

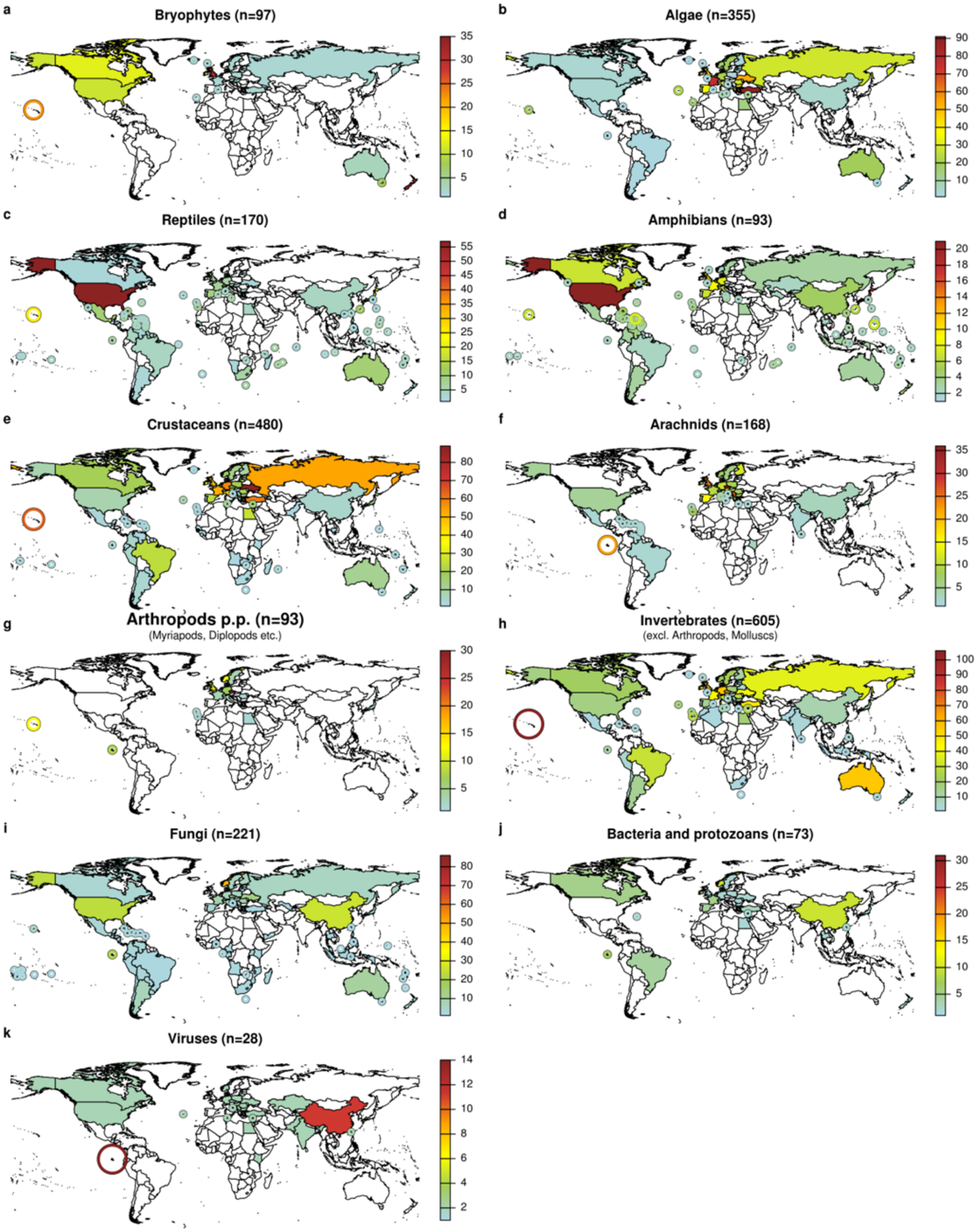

Supplementary Fig. 1 Number of first records of established alien species per region (mainlands and islands) for taxonomic groups with a low number of first records (a-k).

Colour and size of circles indicate the number of first records of established alien species. Circles denote first records on small islands and archipelagos otherwise not visible. The world maps were created using the ‘maptools’ package[1] of the open source software R[2].

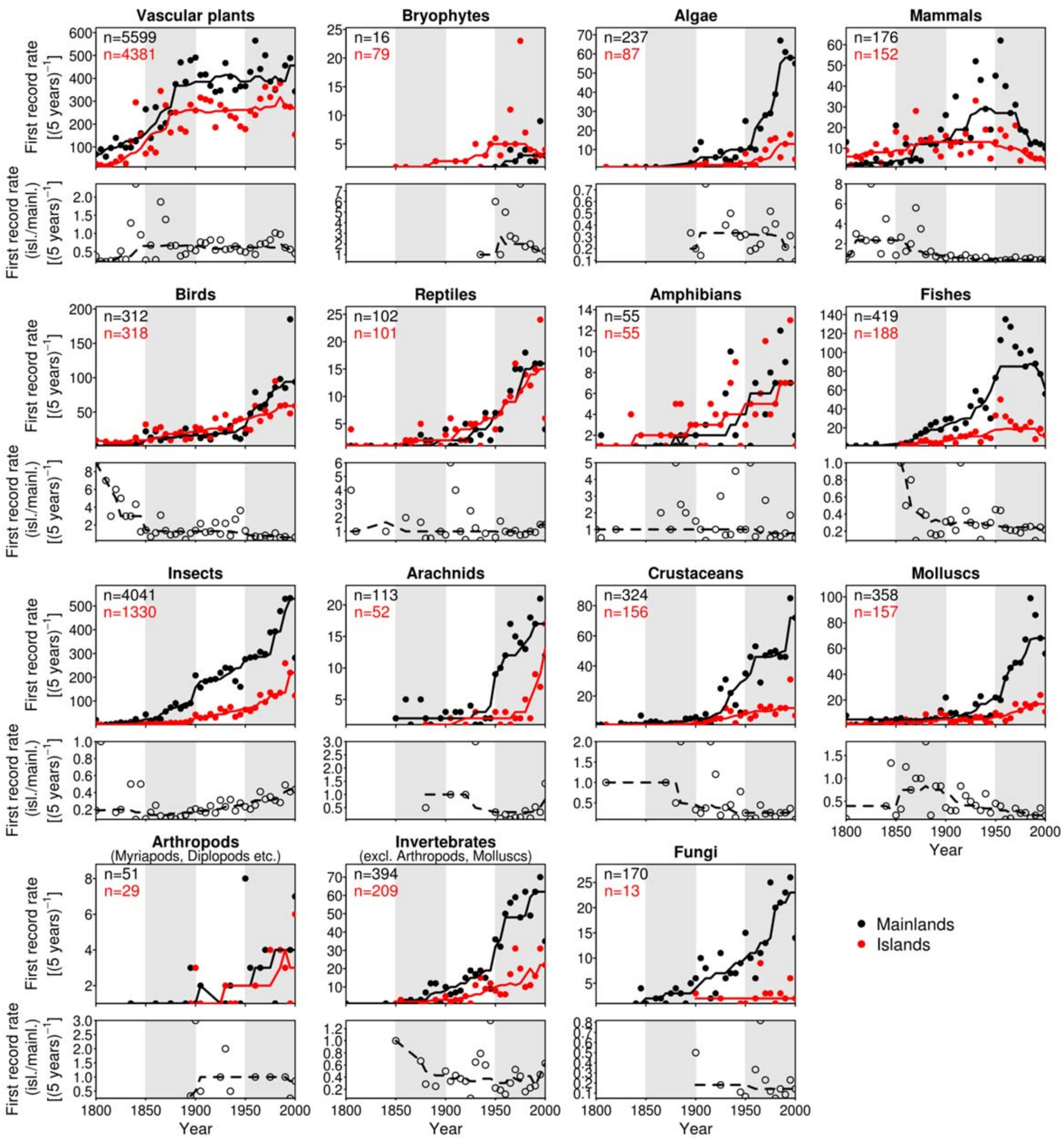

Supplementary Fig. 2 Comparison of first record rates on islands (red) and mainlands (black) for various taxonomic groups. First record rates constitute the number of first records of alien species within a period of five years. Trends are indicated by a running median with a window of 15 data points (lines). The number of established alien species considered on islands or mainlands are given in the upper left of the sub-panels in the respective colour. The ratio of

island to mainland first record rates is shown below the original time series (circles). A decline of the ratio indicates a stronger increase of first record rates on mainlands compared to islands.

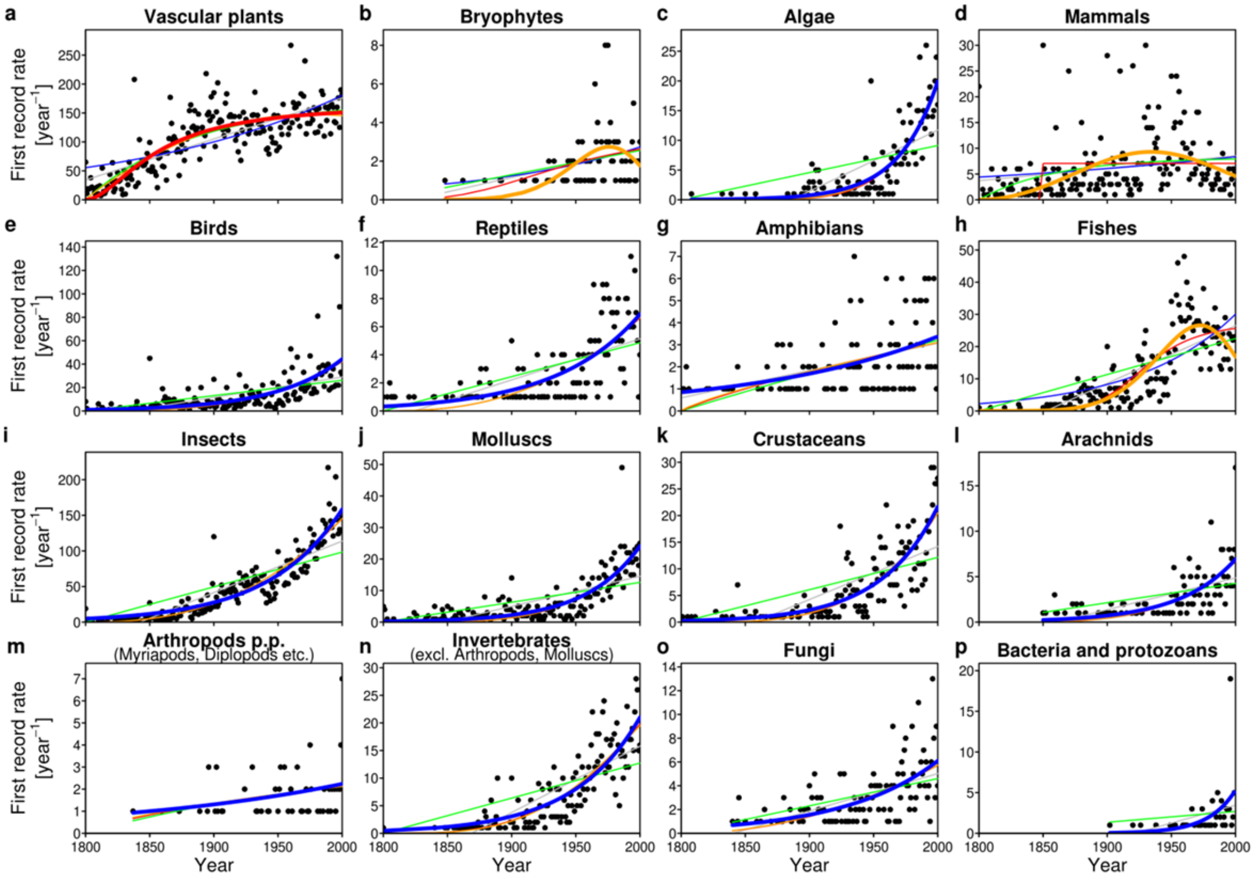

Supplementary Fig. 3 Trends (lines) in the temporal development of first record rates (dots) of taxonomic groups. To mathematically describe the trends, different functions were fitted to the time series' of first record rates and the best-fitting function indicated by the lowest AIC was selected (highlighted as a bold line). The relationships between time and first record rates were described using the following functions: linear (grey), exponential (blue), saturating (green), sigmoidal (red), hump-shaped (orange).

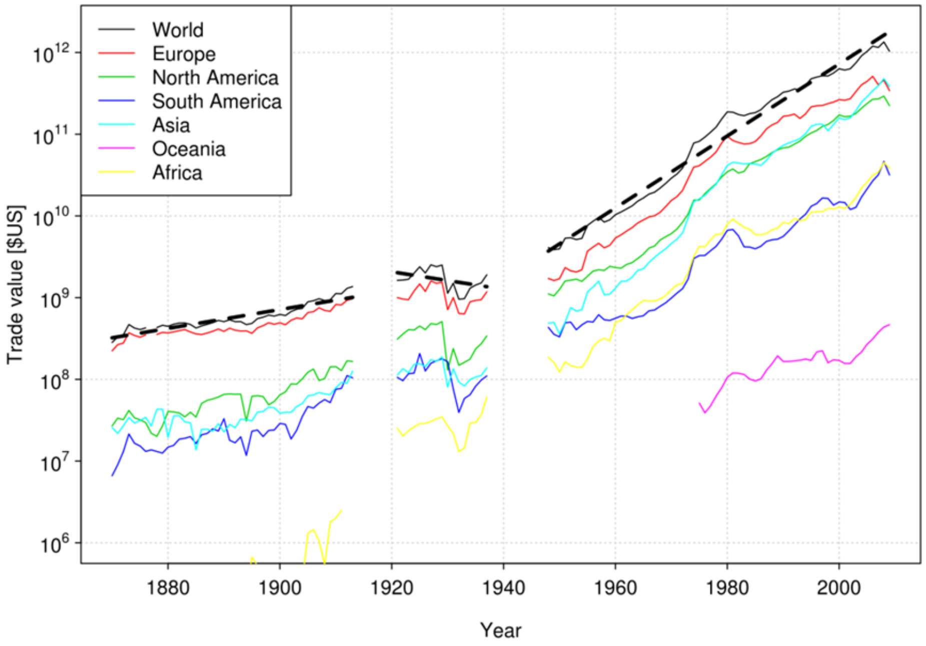

Supplementary Fig. 4 Temporal trends of the sum of annual values of imported commodities of all countries of the world (black) and of all countries of a continent (colours) in current US$ from 1870 to 2009. The trend is indicated by a linear model on a semi-logarithmic scale for global imports (dashed line) fitted separately to import values of 1870-1914, 1920-1938, 1948-2009, respectively. Gaps indicate a lack of data due to the World Wars.

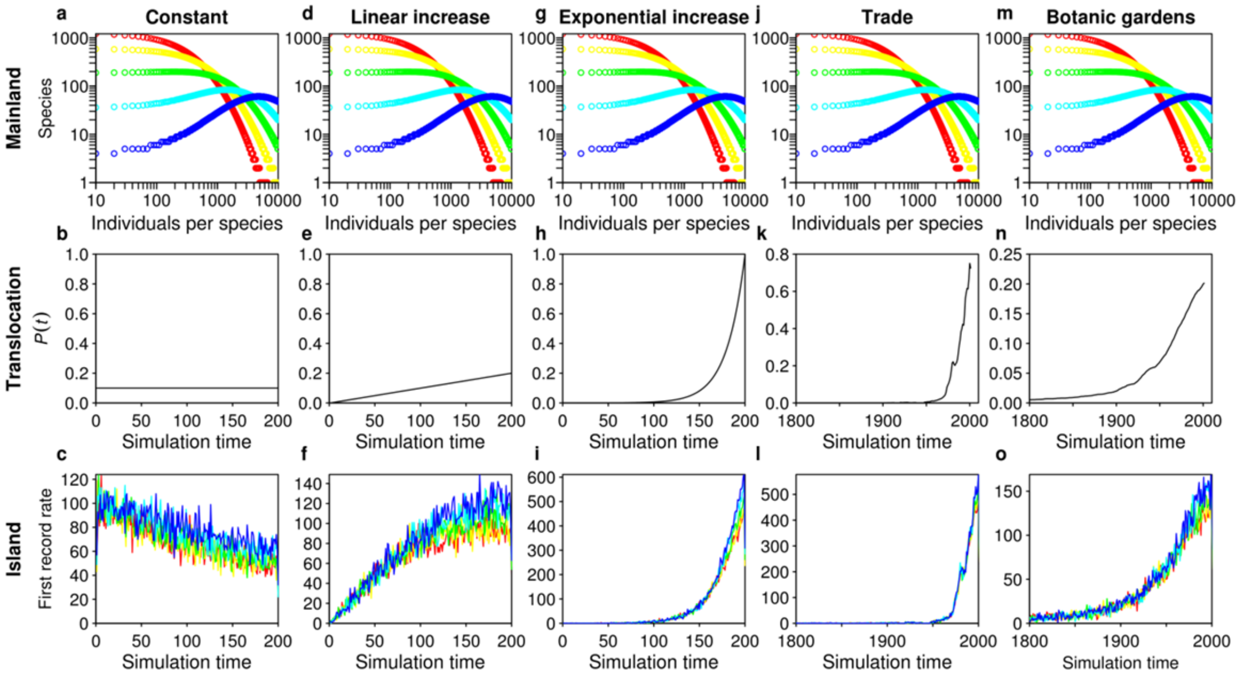

Supplementary Fig. 5 Simulation results of the invasion process from a mainland community (upper panels) to an island (lower panels) for various shapes of the temporal development of the probability of translocation (middle panels). (a, d, g, j, m) Five arbitrary mainland communities (colours) with the same number of log-normally distributed species (n=50,000) but varying relative contributions were considered. (b, e, h, k, n) At each simulation time step *t*, a propagule from one of the mainland communities was selected with probability $P(t)$ and translocated to the island. The temporal development of $P(t)$ was assumed to be constant (b), to increase linearly (e) or exponentially (h), or to be proportional to the global annual value of imported commodities (k) or proportional to the cumulative number of founded botanic gardens (n). (c, f, i, l, o) The resulting first record rates on the island depend on the shape of the temporal development $P(t)$ while the contribution of species in the mainland community has little influence (see Supplementary Notes for more details).

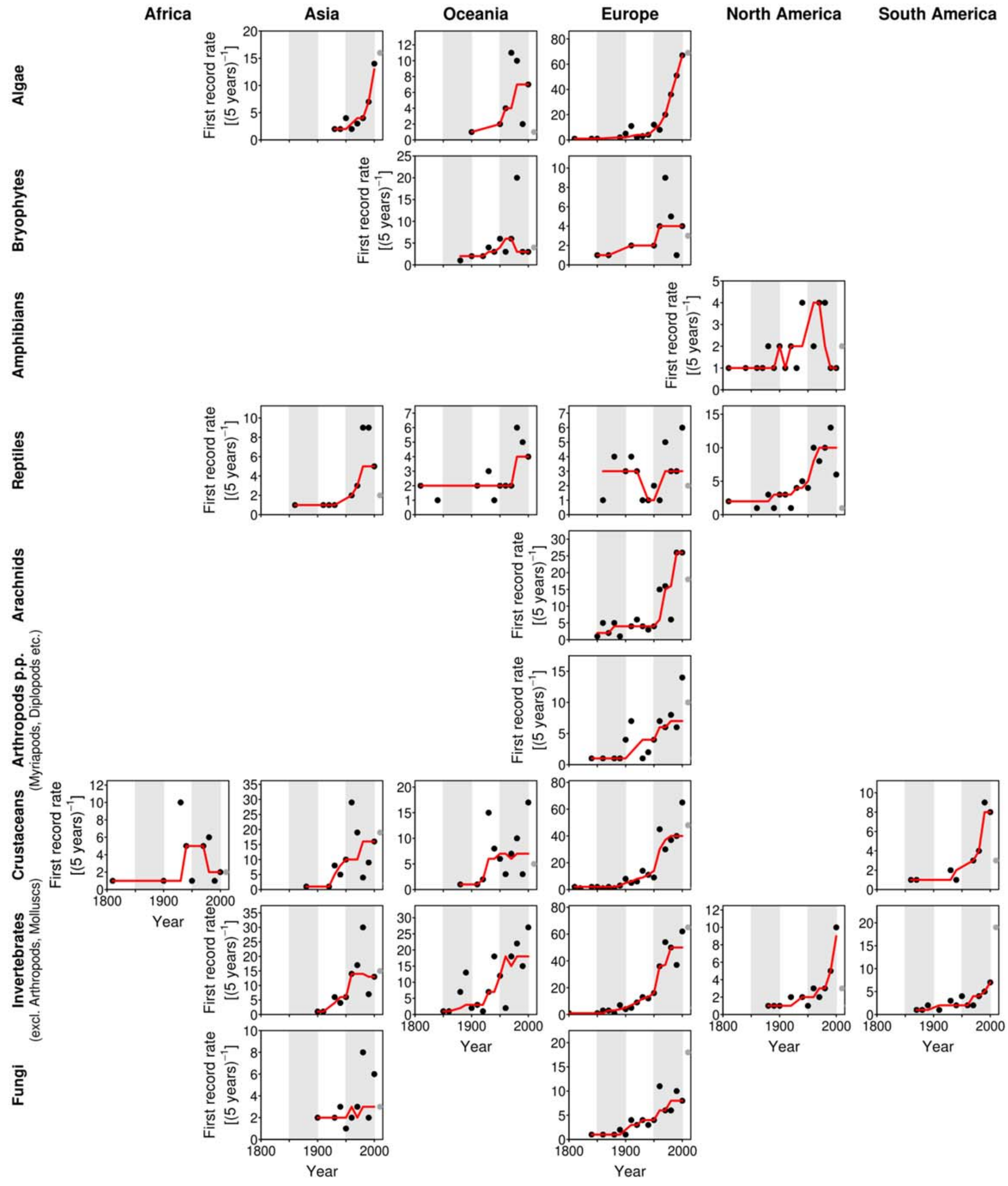

Supplementary Fig. 6 Temporal trends of continental first record rates (i.e., first records of established alien species on a continent per ten years, dots) for various taxonomic groups with low numbers of first records per continent (for delineation of continents see

Supplementary Fig. 12). The trend is indicated by a running median with 50-year moving window (red line). Data after 2000 (grey dots) are incomplete and were removed from analysis. Time series' with less than 30 first records are not plotted. For visualization, 50-year periods are distinguished by white/grey shading.

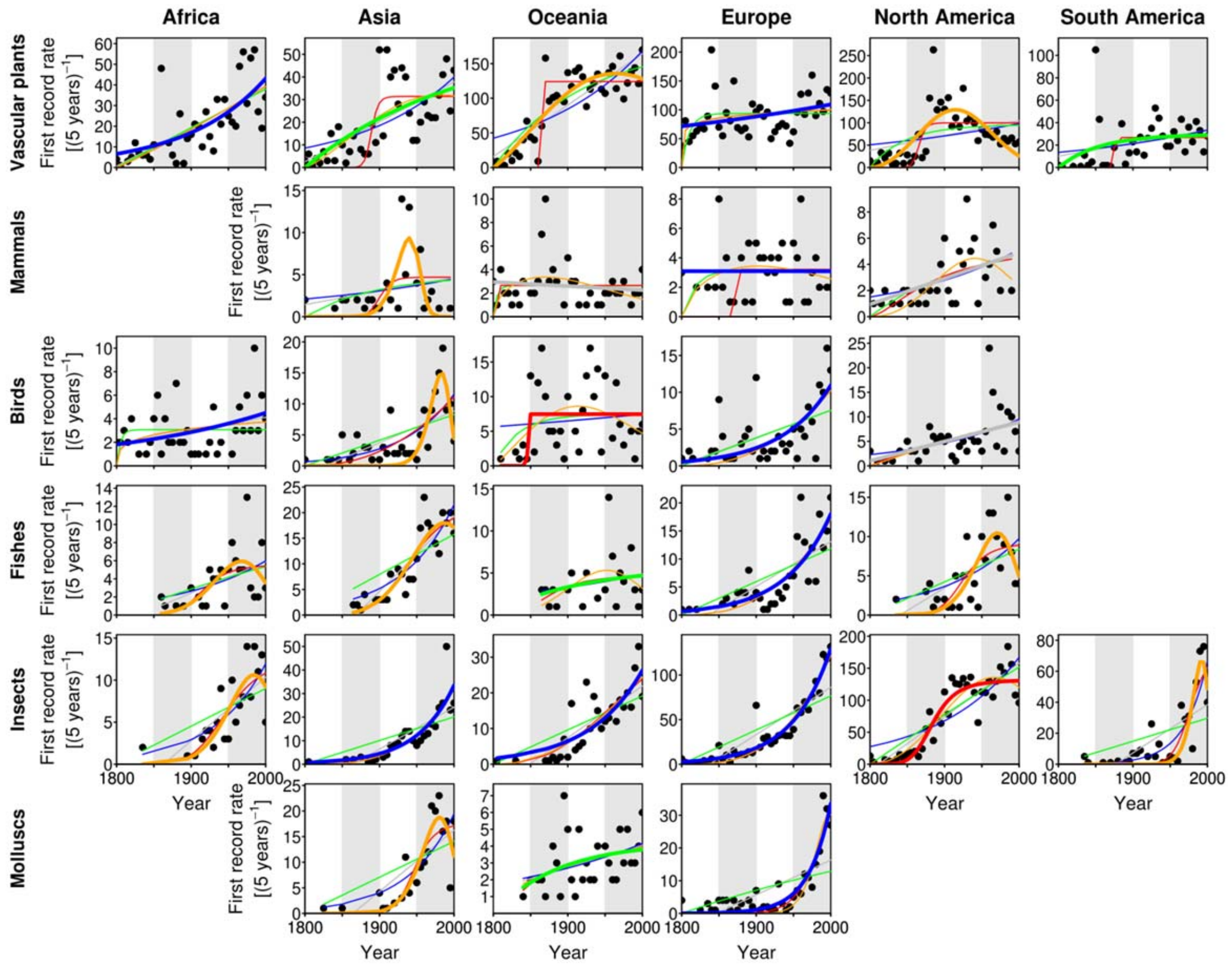

Supplementary Fig. 7 Trends (lines) in the temporal development of first record rates (dots) of taxonomic groups. To mathematically describe the trends, different functions were fitted to the time series' of first record rates and the best-fitting function indicated by the lowest AIC was selected (highlighted in bold). The relationships between time and first record rates were described using the following functions: linear (grey), exponential (blue), saturating (green), sigmoidal (red), hump-shaped (orange). In many cases (10 out of 30), the hump-shaped function was the best-fitting model; however, often the declining trends, which led to the good fit of this type of function, were due to a few low values at the very end of the time series, which are likely to be affected by a delay in recognition and reporting of new alien species (e.g., insects

in Africa or South America). In these cases, the application of a hump-shaped function is not well justified as the fit did only marginally perform better than other functions.

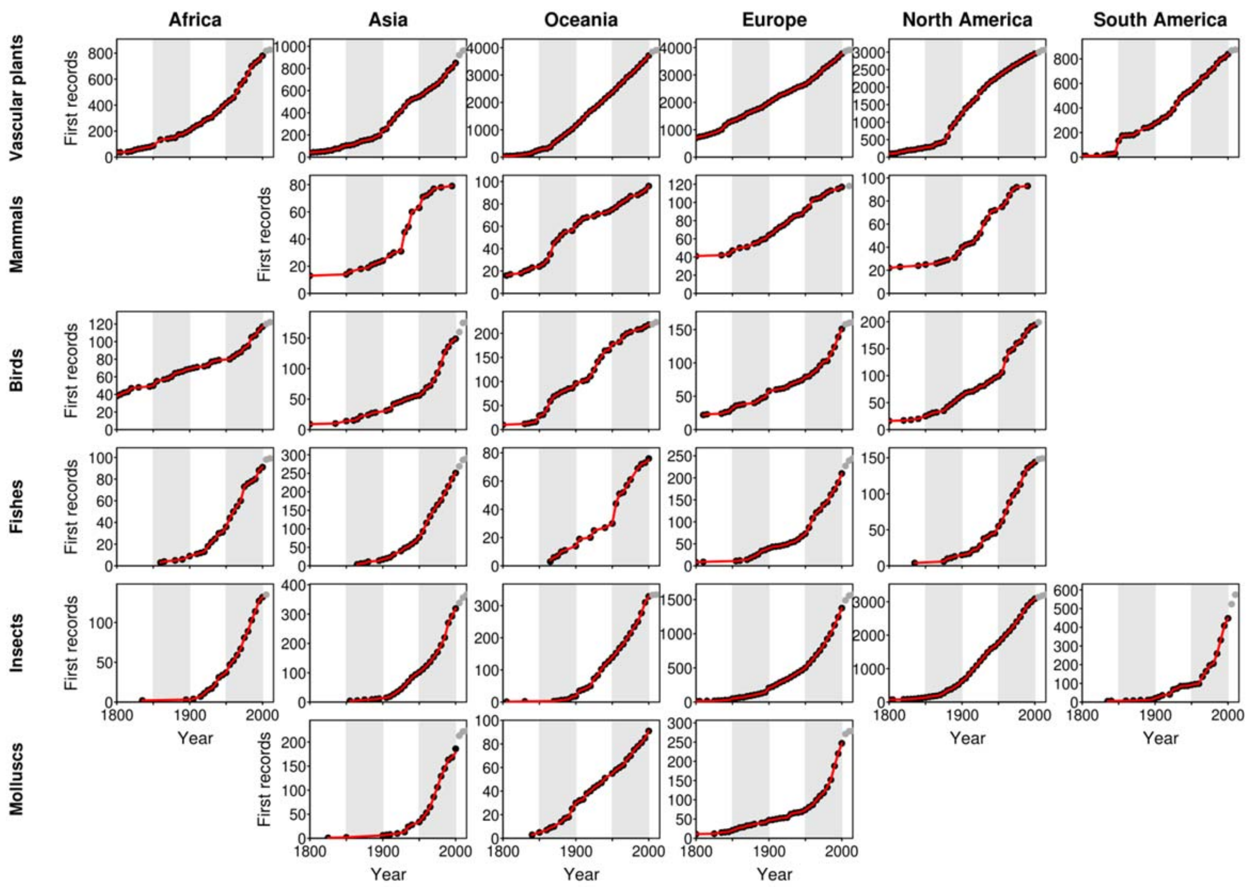

Supplementary Fig. 8 Cumulative numbers of alien species on a continent (dots) for various taxonomic groups (for delineation of continents see Supplementary Fig. 12). The record of an alien species was only considered once on a continent. The trend is indicated by a running median with 25-year moving window (red line). Data after 2000 (grey dots) are incomplete and not considered for analysis. For visualization, 50-year periods are distinguished by white/grey shading.

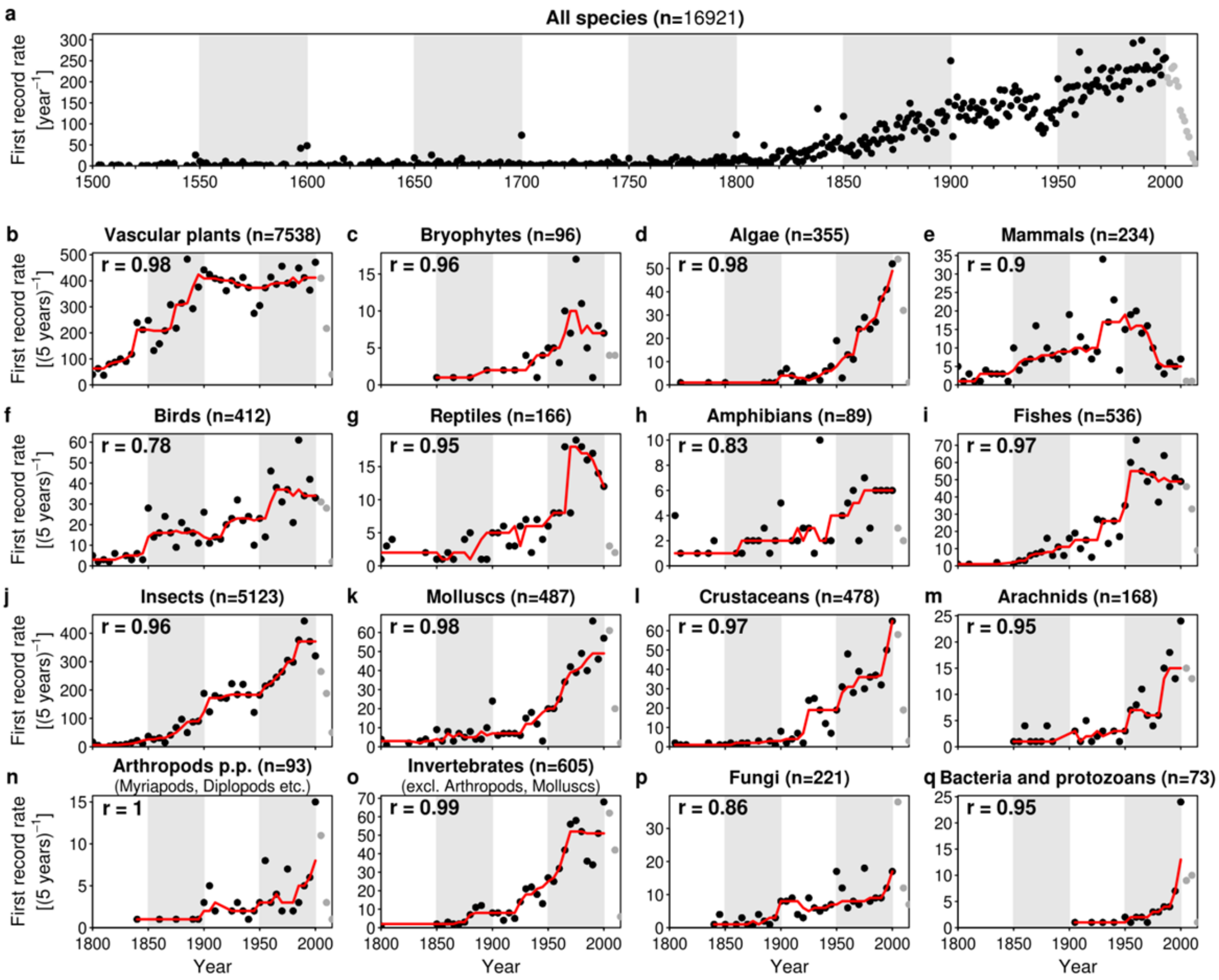

Supplementary Fig. 9 Temporal development of first record rates defined as first record of an established alien species on a continent for (a) all species and (b-q) various taxonomic groups (for delineation of continents see Supplementary Fig. 12). The consideration of first records of alien species on a continent removes a large amount of the influence of secondary invasions (i.e., the subsequent spread after first introduction). This reduces the number of first records and increases the amount of noise in the time series. The cross-correlation coefficients with time lag zero between the time series' of first records on a continent and first records in a mainland country/island (Fig. 2 in the main text) are shown in the upper left corner to enable an assessment of the similarity of both time series. 81% of all coefficients are >0.9 and all >0.78,

which indicates a high similarity of first record rates on both geographic scales. For visualization, 50-year periods are distinguished by white/grey shading.

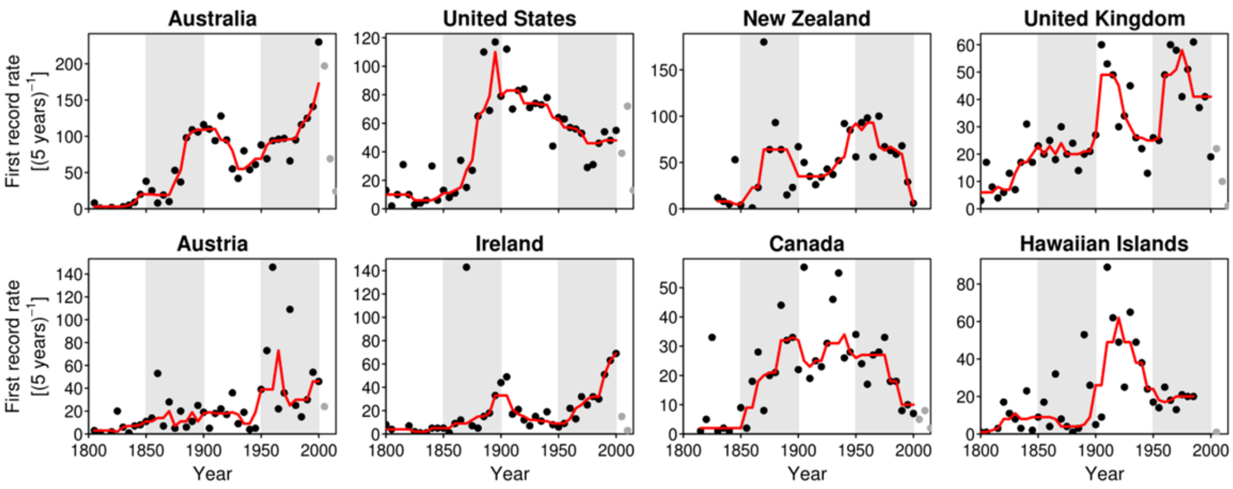

Supplementary Fig. 10 Temporal trends of first record rates of vascular plants of countries with highest numbers of first records of vascular plants. The trend is indicated by a running median with 25-year moving window (red line). Data after 2000 (grey dots) are incomplete because of the delay between sampling and publication and therefore not included in the analysis. First record rates constitute the number of first records per five years (dots) during 1800-2014. For visualization, 50-year periods are distinguished by white/grey shading.

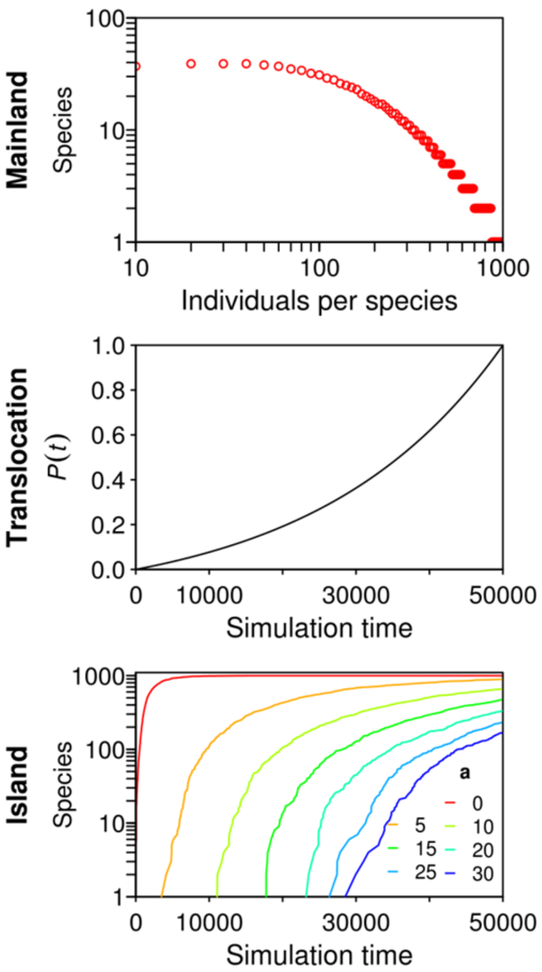

Supplementary Fig. 11 Simulation results of the accumulation of alien species using the extended model version with the consideration of an Allee effect. From an arbitrary mainland community of 1,000 species with log-normally distributed abundances (a) a single propagule is introduced to an island with probability $P(t)$ at each time step t, while $P(t)$ increased with time as shown in (b). On the island, the alien population is considered to become established if the number of propagules of the same species found during a period T on the island exceeds the Allee threshold a. (c) Alien species accumulation curves for various values of a ranging from 0 (red) to 30 (blue) propagules. Increasing a resulted in a more delayed accumulation of alien species, while the overall shape of the accumulation curves remained similar. Note that the

implementation of the Allee effect introduced a new source of stochasticity to the model, which resulted in slightly fluctuating invasion curves.

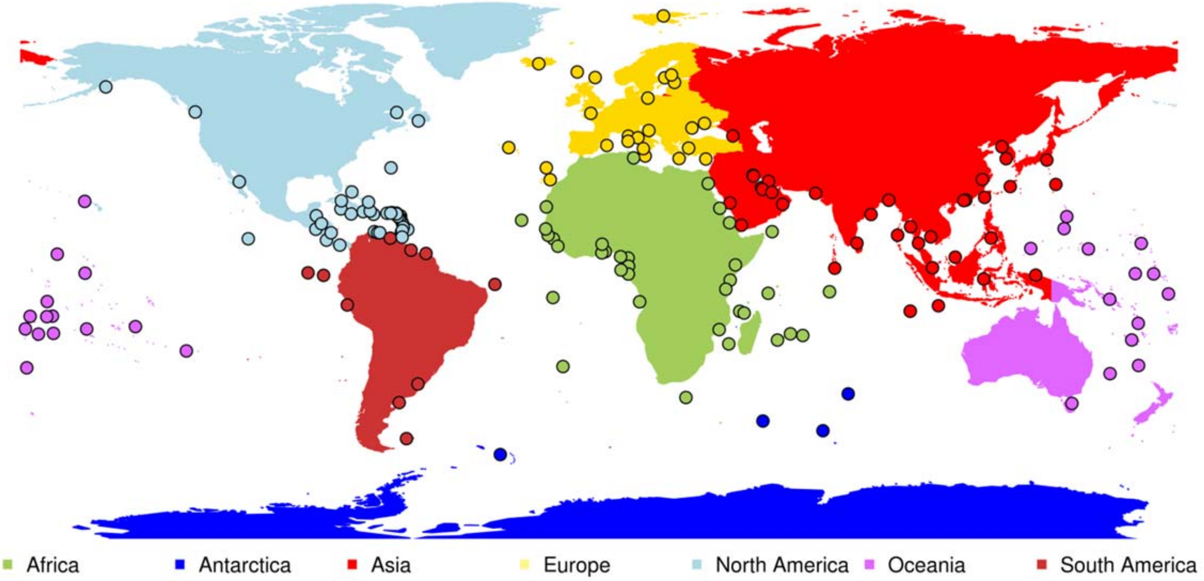

Supplementary Fig. 12 Delineation of continents (colours) used for analysis. The delineation of continents oriented at political borders of countries modified by biogeographical boundaries for islands. Islands (dots) were assigned to the nearest continent depending on their biogeographical location irrespective if they were politically part of a mainland country. For example, Hawaiian Islands (United States) were considered as Islands of Oceania and La Réunion (France) as an African Island. The world map was created using the ‘maptools’ package[1] of the open source software R[2].

Supplementary Tables

Supplementary Table 1 List of sources of first records of alien species used for the compilation of the database. The number of records denotes the number of species considered in this study and may be higher in the original source. In some cases, more data could be obtained from the authors or from the data providers for this study than provided online or in the literature.

**Online databases**		
No	No of records	Database
1	8721	Delivering Alien Invasive Species Inventories for Europe (DAISIE) (http://www.europe-aliens.org/)
2	3219	Global Invasive Species Database (GISD), Invasive Species Specialists Group (ISSG) (http://www.issg.org/database/welcome/aboutGISD.asp)
3	2878	Australia Virtual Herbarium, Council of Heads of Australasian Herbaria (http://avh.chah.org.au/)
4	1864	CABI Invasive Species Compendium, Wallingford , UK: CAB International (http://www.cabi.org/isc/)
5	1458	FishBase, R. Froese and D. Pauly (Editors) 2015. World Wide Web electronic publication. (http://www.fishbase.de/
6	857	Collection from herbaria of North-West North America, provided by Alexander Mosena:<br>Rocky Mountain/US Forest Service herbaria, Laramie, Wyoming, USA (http://www.rmh.uwyo.edu/about/policies.php)
		Oregon Flora Project. 2013. OSU Herbarium specimen data with Oregon Flora Project nomenclature. Oregon State University Herbarium, Corvallis, Oregon, USA; Retrieved 12 September 2013 (http://oregonflora.org)
		University of British Columbia Herbarium, Beaty Biodiversity Museum, Vancouver, British Columbia, Canada (http://www.beatymuseum.ubc.ca/herbarium)
		Wesley E. Niles Herbarium, University of Nevada, Las Vegas, Nevada, USA (https://www.unlv.edu/lifesciences/herbarium)
7	749	Aquatic Non-Indigeneous and Cryptogenic Species Database (AquaNIS). Editorial Board, 2015. Information system on Aquatic Non-Indigenous and Cryptogenic Species. World Wide Web electronic publication. Version 2.36+ (www.corpi.ku.lt/databases/aquanis)
8	730	Consortium of California Herbaria (http://ucjeps.berkeley.edu/consortium/); data provided by David Baxter

9	669	CONABIO (2013) Sistema de información sobre especies invasoras en México. Comisión Nacional para el Conocimiento y Uso de la Biodiversidad (http://www.conabio.gob.mx/invasoras)
10	627	Introduced Species of Galapagos, Charles Darwin Foundation (http://www.darwinfoundation.org/datazone/checklists);<br>Bungartz, F., Herrera, H.W., Jaramillo, P., Tirado, N., Jiménez-Uzcátegui, G., Ruiz, D., Guézou, A. & Ziemmeck, F. (eds.) (2009). Charles Darwin Foundation Galapagos Species Checklist - Lista de Especies de Galápagos de la Fundación Charles Darwin. Charles Darwin Foundation / Fundación Charles Darwin, Puerto Ayora, Galapagos: http://www.darwinfoundation.org/datazone/checklists/ Last updated 09 Nov 2015.<br>Data provided by Heinke Jäger
11	93	Asian-Pacific Alien Species Database (APASD), National Institute for Agro-Environmental Sciences, Japan (http://www.niaes.affrc.go.jp/techdoc/apasd/menu.html)
12	54	The Biota of North America Program (BONAP, http://www.bonap.org/); data provided by Dr. John T. Kartesz and Misako Nishino
13	46	Alien Species of Japan, National Institute for Environmental Studies, Japan (https://www.nies.go.jp/biodiversity/invasive/index_en.html); data checked and provided by Piero Genovesi and Shyama Pagad
14	2	USDA Natural Resources Conservation Service, United States Department for Agriculture (http://plants.usda.gov)
15	1	Atlas of Living Australia, National Research Infrastructure for Australia (http://biocache.ala.org.au)
16	1	The University and Jepson Herbaria, University of California, Berkeley, USA (http://ucjeps.berkeley.edu)
17	1	Food and Agriculture Organisation (FAO) Corporate Document Repository, World Wide Web electronic publication (http://www.fao.org/wairdocs/ilri/x5491e/x5491e0c.htm)
18	1	The Belgian Forum on Invasive Species (BFIS) (http://ias.biodiversity.be/definitions)

Scientific publications/books/book chapters/reports (in alphabetical order)

No	No of records	Reference
19	327	Aukema, J. E. *et al.* Historical Accumulation of Nonindigenous Forest Pests in the Continental United States. *Bioscience* **60,** 886–897 (2010).
20	188	Barina, Z., Rakaj, M., Somogyi, G., Erős-Honti, Z. & Pifkó, D. The alien flora of Albania: history, current status and future trends. *Weed Res* **54,** 196–215 (2014).
21	23	Barker, G. M. Naturalised terrestrial Stylommatophora (Mollusca: Gastropoda). *Fauna of New Zealand* **38,** 1–253 (1999).
22	11	Barua, S. P., H.Khan, M. M. & Reza, A. H. M. *The Status of Alien Invasive Species in Bangladesh and their Impact on the Ecosystems*. (IUCN-The World Conservation Union, 2000).
23	86	Beenken, L.; Senn-Irlet, B., 2016: Neomyceten in der Schweiz. Stand des Wissens und Abschätzung des Schadpotentials der mit Pflanzen assoziierten gebietsfremden Pilze.

WSL Ber. 50: 92pp. PDF Download www.wsl.ch/publikationen/pdf/15783.pdf

24	231	Brundu, G. & Camarda, I. The Flora of Chad: a checklist and brief analysis. *PhytoKeys* **23,** 1–18 (2013); data provided by Guiseppe Brundu
25	249	Carlton, J. T. & Eldredge, L. G. Marine Bioinvasions of Hawaiʻi - The Introduced and Cryptogenic Marine and Estuarine Animals and Plants of the Hawaiian Archipelago. *Bish Museum Bull Cult Environ Stud* **4,** 1–203 (2009).
26	41	Castro, S. A., Figueroa, J. A., Muñoz-Schick, M. & Jaksic, F. M. Minimum residence time, biogeographical origin, and life cycle as determinants of the geographical extent of naturalized plants in continental Chile. *Divers Distrib* **11,** 183–191 (2005).
27	s. Ref 96	Celesti-Grapow, L. *et al.* The inventory of the non-native flora of Italy. *Plant Biosyst* **143,** 386–430 (2009) (First records provided as a personal collection).
28	2	Chapuis, J. L., Boussès, P. & Barnaud, G. Alien mammals, impact and management in the French subantarctic islands. *Biol Conserv* **67,** 97–104 (1994).
29	135	Çinar, M. E., Bilecenoğlu, M., Öztürk, B., Katagan, T. & Aysel, V. Alien species on the coasts of Turkey. *Mediterr Mar Sci* **6,** 119–146 (2005).
30	36	Coles, S. L., DeFelice, R. C., Eldredge, L. G. & Carlton, J. T. Historical and recent introductions of non-indigenous marine species into Pearl Harbor, Oahu, Hawaiian Islands. *Mar Biol* **135,** 147–158 (1999).
31	29	Cowie, R. H. Patterns of introduction of non-indigenous non-marine snails and slugs in the Hawaiian Islands. *Biodivers Conserv* **7,** 349–368 (1998).
32	105	Essl, F., Steinbauer, K., Dullinger, S., Mang, T. & Moser, D. Telling a different story: a global assessment of bryophyte invasions. *Biol Invasions* **15,** 1933–1946 (2013).
33	252	Frenot, Y., Gloaguen, J. ., Massé, L. & Lebouvier, M. Human activities, ecosystem disturbance and plant invasions in subantarctic Crozet, Kerguelen and Amsterdam Islands. *Biol Conserv* **101,** 33–50 (2001).
34	2215	Gatehouse, H. *Ecology of the naturalisation and geographic distribution of the non-indigenous seed plant species of New Zealand* (PhD. thesis, Lincoln University, New Zealand, 2009); data provided by Philip E. Hulme, Lincoln University, New Zealand.
35	214	Henderson, L. Comparisons of invasive plants in southern Africa originating from southern temperate, northern temperate and tropical regions. *Bothalia* **36,** 201–222 (2006).
36	26	Herbert, D. G. The introduced terrestrial Mollusca of South Africa. *SANBI Biodivers Ser* **15,** 1–117 (2010).
37	85	Hewitt, C. L. *et al.* Introduced and cryptogenic species in Port Phillip Bay, Victoria, Australia. *Mar Biol* **144,** 183–202 (2004).
38	14	Huang, D., Haack, R. A. & Zhang, R. Does global warming increase establishment rates of invasive alien species? A centurial time series analysis. *PLoS One* **6,** e24733 (2011).
39	78	Jaryan, V., Uniyal, S. K., Gupta, R. C. & Singh, R. D. Alien flora of Indian Himalayan state of Himachal Pradesh. *Environ Monit Assess* **185,** 6129–53 (2013).
40	818	Julien, M. H. & Griffiths, M. W. *Biological control of weeds: a world catalogue of agents and their target weeds*. (CAB International, 1999); data provided by Sven Bacher.

41	37	Kiritani, K. & Morimoto, N. Invasive Insect and Nematode Pests from North America. *Glob Environ Res* **8,** 75–88 (2004).
42	474	Kraus, F. *Alien Reptiles and Amphibians - a Scientific Compendium and Analysis*. (Springer Netherlands, 2009).
43	10	Kumar, A. B. Exotic fishes and freshwater fish diversity. *Zoo's Print J* **15,** 363–367 (2000).
44	762	Lavoie, C., Saint-Louis, A., Guay, G. & Groeneveld, E. Les plantes vasculaires exotiques naturalisées : une nouvelle liste pour le Québec. *Le Nat Can* **136,** 6 (2012).
45	49	Lazkov, G. A. & Sultanova, B. A. *Checklist of vascular plants of Kyrgyzstan, Norrlinia 24 (in Russian)*. (Finnish Museum of Natural History, 2011).
46	759	Long, J. L. *Introduced Mammals of the World-Their History, Distribution and Influence*. (CSIRO Publishing, 2003); data provided by Sven Bacher.
47	381	Maroyi, A. The casual, naturalised and invasive alien flora of Zimbabwe based on herbarium and literature records. *Koedoe* **54,** 1–6 (2012).
48	23	Mattson, W. J., Niemela, P., Millers, I. & Inguanzo, Y. *Immigrant Phytophagous Insects on Woody Plants in the United States and Canada: An Annotated List*. (US Department of Agriculture, 1994).
49	592	Medvecká, J. *et al.* Inventory of the alien flora of Slovakia. *Preslia* **84,** 257–309 (2012).
50	4	Miles, J. W., Maass, B. L., do Valle, C. B. & Kumble, V. (eds. . *Brachiaria: Biology, Agronomy, and Improvement*. (Centro Internacional de Agricultura Tropical (CIAT), 1996).
51	49	Mills, E. L., Leach, J. H., Carlton, J. T. & Secor, C. L. Exotic Species in the Great Lakes: A History of Biotic Crises and Anthropogenic Introductions. *J Great Lakes Res* **19,** 1–54 (1993).
52	317	Nealis, V.G. *et al*. Historical occurrence of alien arthropods and pathogens on trees in Canada. Canada J. For. Res. **180**, 1–41 (2016).
53	37	Nehring, S. & Rabitsch, W. in *Naturschutzfachliche Invasivitätsbewertungen für in Deutschland wild lebende gebietsfremde Wirbeltiere (in German)* (eds. Nehring, S., Rabitsch, W., Kowarik, I. & Essl, F.) 222 (Bundesamt für Naturschutz, 2015).
54	76	O'Flynn, C., Kelly, J. & Lysaght, L. Ireland's invasive and non-native species – trends in introductions. *Natl Biodivers Data Cent Ser* **2,** 1–50 (2014).
55	s. Ref 70	Pearman, D.A. & Preston, C.D. *First Records of Alien Plants in the wild in Britain and Ireland*. (London: B.S.B.I., 2003). Data included in Roy et al. (2012).
56	262	Peck, S., Heraty, J., Landry, B. & Sinclair, B. Introduced insect fauna of an oceanic archipelago: The Galápagos Islands, Ecuador. *Am Entomol* **Winter,** 218–237 (1998).
57	10	Peltanová, A., Petrusek, A., Kment, P. & Juřičková, L. A fast snail's pace: colonization of Central Europe by Mediterranean gastropods. *Biol Invasions* **14,** 759–764 (2012).
58	39	Peña (ed.), J. 2013. Potential Invasive Pests of Agricultural Crops. University of Florida, USA.
59	45	Petrova, A., Vladimirov, V. & Georgiev, V. Invasive Alien Species of Vascular Plants in Bulgaria. Institute of Biodiversity and Ecosystem Research, Bulgarian Academy of Sciences Sofia, Sofia. 320pp. (2013)
60	172	Pickard, J. Exotic Plants on Lord Howe Island: Distribution in Space and Time, 1853-1981. *J Biogeogr* **11,** 181–208 (1984).

61 8 Protopopova, V. V. & Shevera, M. V. Ergasiophytes of the Ukrainian flora. Biodivers. Res. Conserv. **35**, 31–46 (2014).

62 763 Pyšek, P., Danihelka, J., Sádlo, J. & Jr, J. C. Catalogue of alien plants of the Czech Republic: checklist update, taxonomic diversity and invasion patterns. *Preslia* **84,** 155–255 (2012).

63 834 Reynolds, S. C. P. A catalogue of alien plants in Ireland. *Occas Pap Natl Bot Gard Glas* **14,** 1–414 (2002).

64 20 Ricciardi, A. Facilitative interactions among aquatic invaders: is an 'invasional meltdown' occurring in the Great Lakes? *Can J Fish Aquat Sci* **58,** 2513–2525 (2001).

65 837 Rojas-Sandoval, J. & Acevedo-Rodríguez, P. Naturalization and invasion of alien plants in Puerto Rico and the Virgin Islands. *Biol Invasions* **17,** 149–163 (2015).

66 20 Roll, U., Dayan, T., Simberloff, D. & Mienis, H. K. Non-indigenous land and freshwater gastropods in Israel. *Biol Invasions* **11,** 1963–1972 (2009).

67 3751 Roques, A. *et al*. Alien terrestrial arthropod in Europe. BioRisk, **4**, 1–1024 (2010) (Insect first records have been updated in the following citation, Ref 68).

68 s. Ref 67 Roques, A. *et al*. Temporal and interspecific variation in rates of spread for insect species invading Europe during the last 200 years. Biol. Invasions, **18**, 907–920 (2016).

69 132 Roy, H. E. *et al.* GB Non-native Species Information Portal: documenting the arrival of non-native species in Britain. *Biol Invasions* **16,** 2495–2505 (2014).

70 1615 Roy, H. E. *et al. Non-Native Species in Great Britain: establishment, detection and reporting to inform effective decision making*. (Non-Native Species Secretariat, 2012).

71 808 Schäfer, H. Chorology and Diversity of the Azorean Flora. *Diss Bot* **374,** 1–130 (2003).

72 297 Šefrová, H. & Laštůvka, Z. Catalogue of alien animal species in the Czech Republic. *Acta Univ Agric Silvic Mendelianae Brun* **3,** 151–170 (2005).

73 87 Senan, A. S., Tomasetto, F., Farcomeni, A., Somashekar, R. K. & Attorre, F. Determinants of plant species invasions in an arid island: evidence from Socotra Island (Yemen). *Plant Ecol* **213,** 1381–1392 (2012).

74 135 Smith, R. M. *et al.* Recent non-native invertebrate plant pest establishments in Great Britain: origins , pathways , and trends. *Agric For Entomol* **9,** 307–326 (2007).

75 290 Stace, C. A. & Crawley, M. J. *Alien Plants*. (William Collins, 2015).

76 145 Sykes, W. R., West, C. J., Beever, J. E. & Fife, A. J. *Kermadec Islands flora: a compilation of modern material about the flora of the Kermadec Islands*. (Manaaki Whenua Press, 2000).

77 51 Tassin, J., Rivière, J.-N., Cazanove, M. & Bruzzese, E. Ranking of invasive woody plant species for management on Réunion Island. Weed Res. **46**, 388–403 (2006).

78 128 Thomas, M. C. *The exotic invasion of Florida. A report on arthropod immigration into the sunshine state*. (2004).

79 21 Tiwari, S., Siwakoti, M., Adhikari, B. & Subedi, K. An Inventory and Assessment of Invasive Alien Plant Species of Nepal, IUCN - The World Conservation Union, Nepal. viii+114 pp. (2005).

80 303 Tokarska-Guzik, B. *The Establishment and Spread of Alien Plant Species (Kenophytes) in the Flora of Poland*. (Wydawnictwo Uniwersytetu Śląskiego, 2005).

81 1822 Verloove, F. Catalogue of neophytes in Belgium (1800-2005). *Scr Bot Belgica* **39,** 1–89 (2006).

82	86	Wan, F.-H. & Yang, N.-W. Invasion and Management of Agricultural Alien Insects in China. Annu. Rev. Entomol. **61**, 77–98 (2016).
83	330	Wasowicz, P., Przedpelska-Wasowicz, E. M. & Kristinsson, H. Alien vascular plants in Iceland: Diversity, spatial patterns, temporal trends, and the impact of climate change. *Flora - Morphol Distrib Funct Ecol Plants* **208,** 648–673 (2013).
84	790	Wester, L. in *Alien plant invasions in native ecosystems of Hawaii: Management and Research* (eds. Stone, C. P., Smith, C. W. & Tunison, J. T.) 99–154 (University of Hawaii Press, 1992).
85	12	Wijesundra, S. Major invasive plant species in different climatic zones of Sri Lanka. in *Proceedings of the national symposium on invasive alien species* 15–21 (2008).
86	20	Williams, S. L. Introduced species in seagrass ecosystems: Status and concerns. *J Exp Mar Bio Ecol* **350,** 89–110 (2007).
87	605	Wu, S. *et al.* Insights of the Latest Naturalized Flora of Taiwan : Change in the Past Eight Years. *Taiwania* **55,** 139–159 (2010).
88	313	Xu, H. *et al.* An inventory of invasive alien species in China. *NeoBiota* **15,** 1–26 (2012).
89	s. Ref 91	Yamanaka, T. *et al.* Comparison of insect invasions in North America, Japan and their Islands. *Biol. Invasions* **17,** 3049–3061 (2015). (First records obtained from Andrew Liebhold and Takehiko Yamanaka)

Providers of personal or unpublished collections

No	No of records	Person and data
90	3426	Ellie Dyer & Tim Blackburn, University College London, London, UK; Data: Global Avian Invasions Atlas (GAVIA); Data: Alien bird first records worldwide
91	2315	Andrew Liebhold, US Forest Service Northern Research Station, Morgantown, WV 26505, USA & Takehiko Yamanaka, National Institute for Agro-Environmental Sciences (NIAES), Tsukuba, 305-8604, Japan; Data: Alien insect first records of North America
92	1012	Franz Essl & Wolfgang Rabitsch (2014) AlienAustria database. Environment Agency, Vienna; Data: Alien vascular plant first records of Austria
93	568	Nicol Fuentes, University of Concepcion, Concepcion, Chile; Data: Alien vascular plant first recordss of Chile and Argentina
94	488	Piero Genovesi, ISPRA (Institute for Environmental Protection and Research), Rome, Italy; Data: Alien mammal first records worldwide
95	418	Silvia Rossinelli & Sven Bacher, University of Fribourg, Switzerland; Data: Alien insect first records worldwide
96	410	Kateřina Štajerová & Petr Pyšek, Institute of Botany AS CR, Czech Republic (unpublished data; see detailed information in Pyšek et al., Ecology 96, 762–774, 2015).
97	563	Laura Celesti-Grapow, Sapienza University, Rome, Italy; Data: Alien vascular plant first records of Italy
98	189	César Capinha, CIBIO/InBIO, Lisboa, Portugal; Data: Alien mammal first records worldwide

99	175	Ingolf Kühn, Helmholtz Centre for Environmental Research - UFZ, Halle, Germany; Data: Alien vascular plant first records of Central Europe; revised after Kühn, I., Klotz, S. 2002. Floristischer Status und gebietsfremde Arten. In: Klotz, S., Kühn, I., Durka, W., (Eds.): BIOLFLOR - Eine Datenbank zu biologisch-ökologischen Merkmalen der Gefäßpflanzen in Deutschland. Schriftenreihe für Vegetationskunde 38: 47-56.
100	62	Margarita Arianoutsou, Ioannis Bazos, Pinelopi Delipetrou, Yannis Kokkoris, Andreas Zikos et al., University of Athens, Greece; Data: Alien vascular plant first records of Greece
101	12	Eckehart Jäger, Martin-Luther-University, Halle-Wittenberg, Germany; Data: Alien vascular plant first records of Mongolia
102	4	Wojciech Solarz, Institute of Nature Conservation, Polish Academy of Sicences, Poland; Data: Alien mammal first records of Poland

Supplementary Table 2 Comparison of the correlation coefficients between the time series of imported commodities and first record rates using deflated or non-deflated import values. Import values were deflated using either the Consumer Price Index (CPI) for the respective countries, which was only available for a short time period (at longest 1969-2000) (obtained from the United States Department of Agriculture Economic Research Service, www.ers.usda.gov), or the CPI of the USA spanning a longer period (1913-2000) (provided by the US Bureau of Labor Statistics and available at www.inflationdata.com) was taken for all countries. For comparison, the non-deflated import values were selected during the same time periods. The mean and the correlation between the correlation coefficients using deflated and non-deflated data are shown for each taxonomic group. The correlation coefficients are lower compared to those presented in Fig. 2 due to the lower sampling size. As the differences of using deflated or non-deflated trade values did only marginally affect the results, we used the non-deflated trade values in the main text because of the longer time period (1870-2000). 'No correlation' indicates that fitting the Michaelis-Menten function revealed a linear relationship parallel to the x-axis. As this resulted in a standard deviation of zero, it was not possible to calculate a correlation coefficient in these cases.

	**CPI of all countries (1969-2000)**		**CPI of USA (1913-2000)**	
	**not deflated**	**deflated**	**not deflated**	**deflated**
Vascular plants	0.266	0.174	0.317	0.328
Bryophytes	0.430	No correlation	0.233	0.231
Algae	0.515	0.521	0.802	0.803
Mammals	No correlation	No correlation	0.141	0.142
Birds	0.297	0.303	0.543	0.557
Reptiles	0.005	0.005	0.656	0.650
Amphibians	0.167	0.162	0.236	0.252
Fishes	0.000	0.064	0.653	0.610
Insects	0.669	0.670	0.763	0.796
Molluscs	0.376	0.372	0.809	0.809
Crustaceans	0.510	0.550	0.653	0.667

Arachnids	0.453	0.485	0.529	0.546
Arthropods p.p. (Myriapods, Diplopods etc.)	0.252	0.291	0.184	0.178
Invertebrates (excl. Arthropods, Molluscs)	0.049	0.058	0.765	0.760
Fungi	0.217	0.219	0.527	0.540
Bacteria and protozoans	0.217	0.219	0.527	0.540
**Mean**	**0.29**	**0.29**	**0.52**	**0.53**
**Correlation**	**0.985**		**0.997**	

Supplementary Notes

Data quality assessment

The analysis of first record rates may be hampered by the availability and quality of data. Sampling intensity varies over time, among geographic regions, and taxonomic groups; thus, the trends of first record rates may be biased by variations in research efforts. The database of first records has a taxonomic prevalence of vascular plants, birds, mammals, fishes, and insects. For vascular plants, the database contains 7,646 species, which is 58% of the total number of established alien plant species globally[3], but many invertebrate groups are under-represented. For bacteria, viruses, and fungi relatively few records are available, which renders an interpretation difficult. Nevertheless, the trends are similar to those observed for invertebrate groups; therefore, this study may serve as a first step towards documenting their global trends in accumulation.

The spatial bias of the database is towards well-sampled regions like North America and Europe, while Africa is under-represented. The temporal trends of first record rates can vary distinctly even between well-sampled continents. For example, while first record rates of alien vascular plants and fishes increased in Europe, they declined (vascular plants) or tend to decline (fishes) in North America (Supplementary Fig. 3). It remains unclear if this is a true pattern or an artefact due to varying sampling intensity or varying delay in reporting new alien species. Other trends such as the decline in first record rate of mammals in recent times are consistent across continents and therefore these trends seemed to be robust and not a result of a spatial bias in our data set. However, in many cases it is difficult to assess a potential influence of a spatial bias. One may assume a higher variability of the first record rates in less sampled regions. Indeed, the

standardised variation of the residuals (expressed as the root-mean-squared-error of first records divided by its mean) of the fits shown in Supplementary Fig. 7 declined with increasing number of first records (Pearson's correlation, $t = -2.23$, df = 34, $p < 0.05$). That is, the residuals of these fits are proportionally lower for regions with more first record data, which can be interpreted as a higher robustness associated with those trends consisting of higher number of first records.

A potential temporal bias is more difficult to assess since historical sampling frequencies are missing in nearly all cases. Some studies of alien vascular plants corrected invasion dynamics using herbarium sampling intensities[4–7]. This may be appropriate if a few native and alien species were compared[4] or if the herbaria could be accessed as a whole, so that additional biases such as the preferential digitalization of new records can be avoided. Otherwise, the consideration of sampling intensities will cause other sampling biases, which have to be additionally addressed. Few studies have applied statistical or mathematical modelling to correct for temporal sampling biases[8–11]. However, without knowledge of the real sampling intensities, the application of these approaches requires strong assumptions, such as a constant propagule pressure, which is not fulfilled in this study.

The consequence of a temporal bias is a change of the time lag between the introduction and detection of new alien species. One may assume a general increase of sampling intensity during the past two centuries. This may have resulted in higher first record rates in early times and a shift to slightly lower slopes of these rates during the whole sampling period, while the average number of alien species should be the same or even higher. However, it is highly unlikely that such a shift can explain the main findings of this study that e.g. first record rates of many

invertebrate groups increased after 1950 more strongly, a decline of mammals and fishes first record rates after 1950, or the distinct increase of vascular plants first record rates in the 19th century.

In summary, our data set is by far the most comprehensive one in terms of geography, taxonomy, and temporal coverage. To account for variations within individual data sets, we pooled data by continents for analyses. Despite the uncertainties mentioned above, the consistency of observed trends also among data sets of different size and quality and within short periods of high sampling intensity, such as the last decades, indicates the overall robustness of the observed trends.

Discussion of model results

The development and application of island-colonization models have a long tradition[12], but a continuous temporal change of the probability of translocation $P(t)$ on islands has rarely been considered. Thus, we first present the simplest cases of a continuous temporal development of $P(t)$, and subsequently increased its complexity for various mainland communities.

In the simplest case, $P(t)$ was set constant for all times. The resulting first record rates on the island were very similar for five different mainland communities, with a steep initial increase and a continuous decline afterwards (Supplementary Fig. 5a-c). A linear increase of $P(t)$ with time resulted in a linear increase of the first record rate with a saturation (Supplementary Fig. 5d-f). Note that a saturation of these rates indicates that alien species numbers still increase but constantly. The resulting temporal dynamics of first record rates were similar to those found for

vascular plants (Fig. 2b), and thus already comparable to observational data. The consideration of an exponential increase of $P(t)$ resulted in an exponential increase of the introduction rate (Supplementary Fig. 5g-i).

To make the model more realistic, we modelled $P(t)$ proportional to the value of imported commodities (in US$); and to incorporate trade data into the model, we linearly interpolated the time series of annual total import values to 1000 values per year for 1800-2000 to allow for 201,000 simulation time steps. As trade values are not available prior to 1870, we set the values from 1800 to 1869 to the same as 1870, which had only negligible effects on the results (i.e., although the intensity of trade before 1870 was assumed to be likely higher than in reality, the rate of new introductions was still around zero, Supplementary Fig. 5l). At each time step, a propagule was selected from the mainland community with probability P proportional to total trade values at that time step. The resulting first record rates were low until around 1950 with a steep increase thereafter (Supplementary Fig. 5j-l). A very similar pattern was found for those taxonomic groups that are most likely introduced through trade such as algae, arachnids, insects, and molluscs (Fig. 2d, j, k, m).

To account for variation of first record rates typical for vascular plants, $P(t)$ was assumed to be proportional to the cumulative number of botanic gardens worldwide. The resulting rates already increased in the 19th century with a further acceleration around 1900 (Supplementary Fig. 5m-o). However, the simulated rates are lower compared to the observed ones particularly in the 19th century, which indicates that additional sources of introduction, such as acclimatization societies,

have to be considered in addition. Nevertheless, even these simple colonization models can reproduce observed invasion dynamics relatively well.

Supplementary References

1. Bivand, R. & Lewin-Koh, N. *maptools: Tools for reading and handling spatial objects*. (R package version 0.8-39, http://CRAN.R-project.org/package=maptools, 2016).
2. R Development Core Team. *R: A language and Environment for Statistical Computing*. (https://www.r-project.org/, R Foundation for Statistical Computing, 2015).
3. van Kleunen, M. *et al.* Global exchange and accumulation of non-native plants. *Nature* **525,** 100–103 (2015).
4. Delisle, F., Lavoie, C., Jean, M. & Lachance, D. Reconstructing the spread of invasive plants: taking into account biases associated with herbarium specimens. *J. Biogeogr.* **30,** 1033–1042 (2003).
5. Dodd, A. J., Burgman, M. A., McCarthy, M. A. & Ainsworth, N. The changing patterns of plant naturalization in Australia. *Divers. Distrib.* **21,** 1038–1050 (2015).
6. Aikio, S., Duncan, R. P. & Hulme, P. E. Lag-phases in alien plant invasions: separating the facts from the artefacts. *Oikos* **119,** 370–378 (2010).
7. Fuentes, N., Ugarte, E., Kühn, I. & Klotz, S. Alien plants in Chile: inferring invasion periods from herbarium records. *Biol. Invasions* **10,** 649–657 (2008).
8. Costello, C. J. & Solow, A. R. On the pattern of discovery of introduced species. *Proc. Natl. Acad. Sci.* **100,** 3321–3323 (2003).
9. Solow, A. R. & Costello, C. J. Estimating the Rate of Species Introductions from the Discovery Record. *Ecology* **85,** 1822–1825 (2004).
10. Belmaker, J., Brokovich, E., China, V., Golani, D. & Kiflawi, M. Estimating the rate of biological introductions: Lessepsian fishes in the Mediterranean. *Ecology* **90,** 1134–1141 (2009).

11. Wonham, M. J. & Pachepsky, E. A null model of temporal trends in biological invasion records. *Ecol. Lett.* **9,** 663–72 (2006).

12. McArthur, R. & Wilson, E. O. *The theory of island biogeography*. (Princeton University Press, 1967).
